# Anti-cancer treatment schedule optimization based on tumor dynamics modelling incorporating evolving resistance

**DOI:** 10.1038/s41598-022-08012-7

**Published:** 2022-03-10

**Authors:** Anyue Yin, Johan G. C. van Hasselt, Henk-Jan Guchelaar, Lena E. Friberg, Dirk Jan A. R. Moes

**Affiliations:** 1 Department of Clinical Pharmacy and Toxicology, Leiden University Medical Center, Albinusdreef 2, 2333 ZA Leiden, The Netherlands; 2 Leiden Network for Personalized Therapeutics, Leiden University Medical Center, Leiden, The Netherlands; 3 Division of Systems Biomedicine and Pharmacology, Leiden Academic Centre for Drug Research (LACDR), Leiden University, Leiden, The Netherlands; 4 Department of Pharmacy, Uppsala University, Uppsala, Sweden

**Keywords:** Cancer therapeutic resistance, Evolutionary theory, Pharmacodynamics, Drug therapy

## Abstract

Quantitative characterization of evolving tumor resistance under targeted treatment could help identify novel treatment schedules, which may improve the outcome of anti-cancer treatment. In this study, a mathematical model which considers various clonal populations and evolving treatment resistance was developed. With parameter values fitted to the data or informed by literature data, the model could capture previously reported tumor burden dynamics and mutant *KRAS* levels in circulating tumor DNA (ctDNA) of patients with metastatic colorectal cancer treated with panitumumab. Treatment schedules, including a continuous schedule, intermittent schedules incorporating treatment holidays, and adaptive schedules guided by ctDNA measurements were evaluated using simulations. Compared with the continuous regimen, the simulated intermittent regimen which consisted of 8-week treatment and 4-week suspension prolonged median progression-free survival (PFS) of the simulated population from 36 to 44 weeks. The median time period in which the tumor size stayed below the baseline level (T_TS<TS0_) was prolonged from 52 to 60 weeks. Extending the treatment holiday resulted in inferior outcomes. The simulated adaptive regimens showed to further prolong median PFS to 56–64 weeks and T_TS<TS0_ to 114–132 weeks under different treatment designs. A prospective clinical study is required to validate the results and to confirm the added value of the suggested schedules.

## Introduction

Emerging treatment resistance during anti-cancer therapy is one of the major causes for cancer patients experiencing treatment failure^1,2^. The occurrence of treatment resistance is mediated by a range of mechanisms^1,2^. Evolutionary mechanisms driven by intra-tumor heterogeneity and the evolving adaptation of tumor cells to the selection pressure of treatment are increasingly acknowledged as a key factor related to the development of treatment resistance^3–7^.

To improve the treatment outcome in cancer patients, it may be important to take the intra-tumor heterogeneity and evolutionary dynamics of tumors into consideration when designing treatment strategies. A clinical genetic biomarker that is useful to capture the tumor heterogeneity and to monitor the evolving treatment resistance in a quantitative way is circulating tumor DNA (ctDNA), i.e. tumor DNA fragments circulating in the bloodstream^2,8–10^. Different from tumor size, which is commonly used as an indicator of anti-cancer treatment effect^11^, ctDNA can be detected from liquid biopsies and allows real-time monitoring with limited patient burden. It has been demonstrated that mutations present in multiple biopsies of primary tumor and metastasis can be detected in ctDNA including those being missed in certain biopsie^12^. In addition, the genetic alternations captured by ctDNA can also be quantified. The relative change of genetic alterations in serial ctDNA analysis could provide important insight into the molecular evolution of the tumor and reveal the mechanisms of resistance to targeted agents^8–10^. Previous studies of ctDNA in colorectal cancer patients have demonstrated a positive selection of mutant *KRAS* clones during epidermal growth factor receptor (EGFR) blockade^10,13^, and a decline in mutant *KRAS* clones upon the withdrawal of the therapy^9^. The concentration of ctDNA has also shown to correlate with tumor burden and stage, and is associated with therapeutic response, such as disease progression and recurrence, in different kinds of cancers^8,9,14–18^.

Monitoring tumor-specific genetic alternations can facilitate selection and adjustment of drugs that target newly developed actionable mutations^2,8^. Such adaptive treatment suppresses the proliferation of resistant tumor clones and thereby overcome or at least delay treatment resistance^2,8^.

Considering evolutionary dynamics, suppressing the emergence of resistance by applying intermittent treatment has also been previously proposed^19,20^. Intermittent treatment allows sensitive cells to utilize their fitness advantage during the withdrawal of treatment to suppress the growth of the resistant population, so that the same treatment can remain effective when it is reinitiated, which is especially relevant in the metastatic setting when cure is not possible^19,21^. This principle was demonstrated in silico with game theory models and with a pilot study of abiraterone in prostate cancer patients^19^. For colorectal cancer, it has been shown that tumor genomes adapt dynamically to intermittent drug schedules and re-challenge of EGFR blockade can be efficient^9^. This strategy is also of emerging clinical interest and has been investigated in several clinical studies^22–27^.

Mathematical modelling and simulation is a widely accepted tool in pharmaceutical research to characterize and understand the interaction among drug treatment, the human body, and disease^11,28–30^. Various mathematical model structures have been used to characterize the tumor dynamics and drug resistance evolution for solid tumors^19,31,32^. Tumor proliferation, regression due to treatment, heterogeneity, and treatment resistance are key elements that are commonly considered in those models^32^. The dynamics of biomarkers can also be incorporated which enables better understanding and prediction of tumor progression^32^. A non-linear mixed-effect modeling approach is commonly applied to account for inter-individual variability (IIV)^32^. Studies developing models for tumor dynamics and evolving drug resistance are mostly aimed at optimizing and individualizing current treatments. Furthermore, they are also aimed at better understanding of emerging drug resistance and identification of outcome predictors^32^. Connecting these models to patients survival and adverse effects with time-to-event modelling is also common to support the understanding of treatment efficacy and enables exploration of optimized dosing schedules^33^. These models could guide the interpretation and clinical decision making process based on observed tumor size dynamics and associated evolution of tumor progression during treatment, and thereby supporting identification of novel personalized strategies to optimize anti-cancer treatment schedules and overcome treatment resistance.

The aim of the current study was to develop a mathematical model to quantitatively characterize the dynamics of treatment response and evolving resistance, based on tumor sizes and mutant *KRAS* levels in ctDNA from metastatic colorectal cancer (mCRC) patients. We also aimed to evaluate anti-cancer treatment designs which consider cancer resistance evolution and demonstrate the use of ctDNA as a marker to guide adaptive treatment. These aspects might be beneficial to improve the treatment outcome especially in the metastatic setting. Data identified from the literature were used for model development. Anti-cancer treatment schedules, including continuous, intermittent, and adaptive schedules guided by ctDNA measurements were designed to evaluate optimal treatment schedules.

## Results

### Data and model evaluation

**Figure 1:**
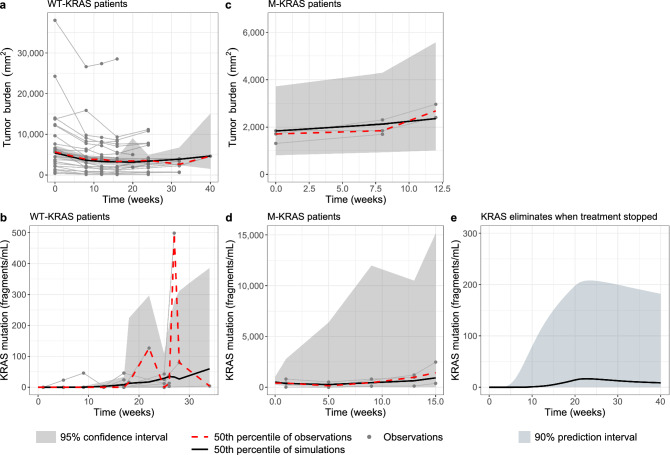
Model evaluation results on the data of tumor burden (**a**, **c**) and mutant *KRAS* (**b**, **d**) collected from a previous clinical trial on patients with metastatic colorectal cancer who were identified to be initially *KRAS* wild-type (**a**, **b**) or had detectable mutant *KRAS* at the start of treatment (**c**, **d**); Model predicted mutant *KRAS* concentrations under a regimen of 20-week treatment and 20-week suspension (**e**).

A dataset containing longitudinal tumor burden measurements and mutant *KRAS* levels in ctDNA was identified from 28 mCRC patients treated with the anti-EGFR inhibitor panitumumab in a previous clinical study^13^ (Fig. 1). Among the 28 patients, 25 were identified to be initially *KRAS* wild-type and 9 of those 25 developed *KRAS* mutation after 5–34 weeks’ (median 22 weeks’) treatment. The remaining 3 patients had detectable mutant *KRAS* at the start of treatment. The characteristics of the patients are summarized in Supplementary Table S1.

**Figure 2:**
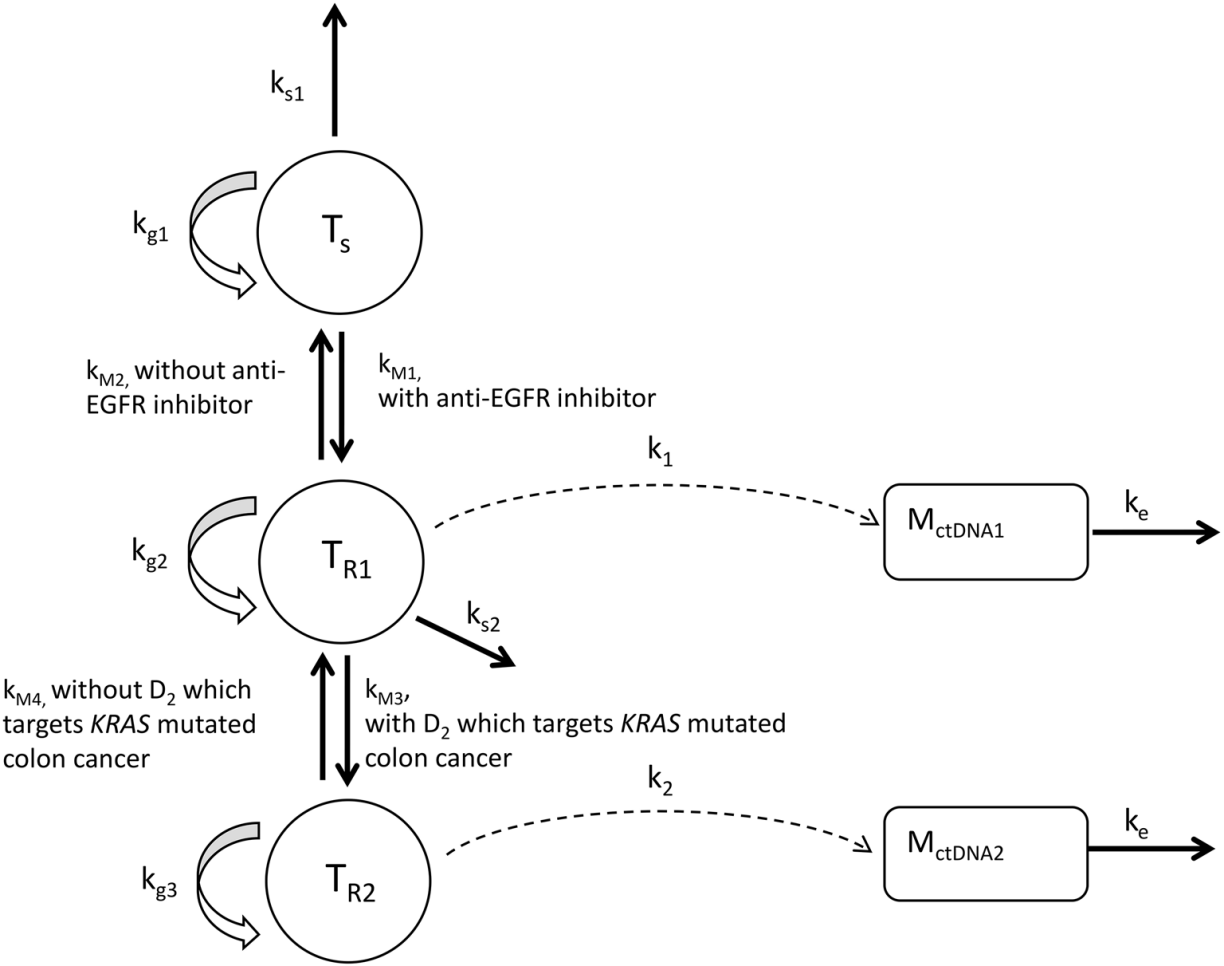
The model that characterizes the dynamics of tumor size and mutation concentrations in ctDNA from metastatic colorectal cancer patients. ${T}_{s}$, ${T}_{R1}$, and ${T}_{R2}$ represent the sizes of three tumor clonal populations, respectively. ${M}_{ctDNA1}$ and ${M}_{ctDNA2}$ represent the concentration of mutant *KRAS* and a hypothetical mutation in ctDNA. ${k}_{g1}$, ${k}_{g2}$, ${k}_{g3}$ represent the net growth rate constants of three clonal populations. ${k}_{s1}$ and ${k}_{s2}$ represent the tumor shrinkage rate due to treatments. ${k}_{M1}$ and ${k}_{M3}$ represent the mutation rate constant from drug susceptible clonal population to drug resistant clonal population during the course of anti-EGFR treatment (${D}_{1}$) and a hypothetical treatment (${D}_{2}$), respectively. ${k}_{M2}$ and ${k}_{M4}$ represent the transition rate constant from drug resistant clonal population to drug susceptible clonal population upon the withdrawal of treatments. ${k}_{1}$ and ${k}_{2}$ represent the shedding rate constant of ctDNA which carries mutations. ${k}_{e}$ represent the elimination rate constant of ctDNA.

The developed model consists of three clonal tumor populations, including $${T}_{s}$$ which was sensitive to anti-EGFR inhibitor ($${D}_{1}$$), $${T}_{R1}$$ which harbored *KRAS* mutation and was resistant to $${D}_{1}$$, and $${T}_{R2}$$ which was resistant to both $${D}_{1}$$ and a hypothetical second treatment targeting $${T}_{R1}$$($${D}_{2})$$, as well as two compartments for mutant *KRAS* ($${M}_{ctDNA1}$$) and a hypothetical second mutation ($${M}_{ctDNA2}$$) in ctDNA (Fig. 2). $${M}_{ctDNA1}$$ and $${M}_{ctDNA2}$$ were assumed to emerge during treatment. Shedding rates of ctDNA depended on the size of $${T}_{R1}$$ and $${T}_{R2}$$, and Hill equations with tumor size as independent variable were applied to describe the delayed emergence (or ability to detect) of mutant genes in ctDNA. Values of model parameters were obtained by fitting to the data or informed by literature (Table 1). Parameters describing tumor dynamics under $${D}_{1}$$ therapy were estimated based on the observed raw data and the results are shown in Supplementary Table S2.

**Table 1:** Parameters values of the developed model characterizing the dynamics of tumor size and mutation concentrations in metastatic colorectal cancer (mCRC) patients.

Parameters (units)	Description	Typical values		Reference
		WT-*KRAS* patients	M-*KRAS* patients	
${T}_{s\_0}$(mm^2^)	Baseline of ${T}_{s}$ (clonal population that is sensitive to anti-EGFR inhibitor (${D}_{1}$) )	5500	100	Data/Estimated value; Mutation was assumed to be acquired during treatment
${T}_{R1\_0}$(mm^2^)	Baseline of ${T}_{R1}$ (clonal population that is resistance to ${D}_{1}$ but is sensitive to the second hypothetical treatment (${D}_{2}$))	0	1700	Data/Estimated value; Mutation was assumed to be acquired during treatment
${T}_{R2\_0}$(mm^2^)	Baseline of ${T}_{R2}$ (clonal population that is resistance to both treatments)	0	0	Data/Estimated value; Mutation was assumed to be acquired during treatment
${M}_{ctDNA1\_0}$(fragments/ml)	Baseline of mutant *KRAS* $\left({M}_{ctDNA1}\right)$ in ctDNA	0	500	Data/Estimated value; Mutation was assumed to be acquired during treatment
${M}_{ctDNA2\_0}$(fragments/ml)	Baseline of a second hypothetical mutation $\left({M}_{ctDNA2}\right)$ in ctDNA	0	0	Data/Estimated value; Mutation was assumed to be acquired during treatment
${k}_{g1}$(/week)	Growth rate constant of ${T}_{s}$	0.03		^40^
${k}_{g2}$(/week)	Growth rate constant of ${T}_{R1}$	0.021		^43,44^
${k}_{g3}$(/week)	Growth rate constant of ${T}_{R2}$	0.015		^43,44^
${k}_{s1}$(/week)	Tumor shrinkage rate constant due to ${D}_{1}$	0.1		Estimated value
${k}_{s2}$(/week)	Tumor shrinkage rate constant due to ${D}_{2}$	0.1		${k}_{s1}$
${k}_{M1}$(/week)	Mutation rate from ${T}_{s}$ to ${T}_{R1}$ when ${D}_{1}$=1	0.05		Estimated value
${k}_{M2}$(/week)	Mutation rate from ${T}_{R1}$ to ${T}_{s}$ when ${D}_{1}$=0	0.03		Lower than ${k}_{M1}$^9^
${k}_{M3}$(/week)	Mutation rate from ${T}_{R1}$ to ${T}_{R2}$ when ${D}_{2}$=1	0.05		${k}_{M1}$
${k}_{M4}$(/week)	Mutation rate from ${T}_{R2}$ to ${T}_{R1}$ when ${D}_{2}$=0	0.03		${k}_{M2}$
$H$	Hill coefficient	5		Visually matching the slope of data and the detectable time of mutant *KRAS*
${KT}_{50}$(mm^2^)	The size of tumor that provide half-maximal shedding rate of ctDNA	3500		Visually matching the slope of data and the detectable time of mutant *KRAS*
${k}_{\mathrm{max}\_1}$((fragments/ml)/(week*mm^2^))	Maximum shedding rate of ${M}_{ctDNA1}$	0.015	1.5	Visually matching the slope of data and the detectable time of mutant *KRAS*
${k}_{e}$(/week)	ctDNA eliminate rate constant	0.5		Visually matching the slope of data and the detectable time of mutant *KRAS*
${k}_{\mathrm{max}\_2}$((fragments/ml)/(week*mm^2^))	Maximum shedding rate of ${M}_{ctDNA2}$	0.015	1.5	${k}_{\mathrm{max}\_1}$
IIV_ $B$ (${\omega }_{1}$)	Standard deviation of IIV of baselines	0.6		Data
IIV_ ${k}_{g}$ (${\omega }_{2}$)	Standard deviation of IIV of ${k}_{g}$	0.2		Data

ctDNA, circulating tumor DNA; IIV, inter-individual variability; WT-KRAS patients, patients who were initially identified as *KRAS* wild-type in ctDNA; M-KRAS patients, patients who had detectable mutant *KRAS* in ctDNA pre-treatment.

The model evaluation results show that the 50th percentiles of the simulated time-courses of total tumor size ($TS$) and mutant *KRAS* (${M}_{ctDNA1}$) concentrations were generally in line with the 50th percentiles of corresponding observations (Fig. 1). The 50th percentiles of observations were also adequately covered by the 95% confidence intervals (CIs) of corresponding percentile obtained from the simulations. Upon a treatment suspension after 20 weeks of treatment, a decay of *KRAS* levels that was observed in previous studies^9^ could also be described by the model. The median and 90% prediction interval of corresponding simulations of 100 virtual patients were shown in Fig. 1E. The predicted median half-life of *KRAS* levels was 4.98 months.

An available dataset on 16 non-small cell lung cancer (NSCLC) patients was utilized as an evaluation cohort (Supplementary Table S3)^14^. Patients included in this study had detectable *EGFR* L858R mutation / exon 19 deletion at the start of treatment and developed *EGFR* T790M mutation during treatment. The model used in the validation cohort was adjusted according to the findings of the study, the details of which can be found in Supplementary method and Fig. S1. Model evaluation results show that the distribution of the model simulations was also in line with the distribution of the tumor size and concentrations of mutant *EGFR* obtained from NSCLC patients (Supplementary Fig. S2).

### Treatment schedule evaluation

Based on the developed model, multiple dosing schedules, including a continuous ${D}_{1}$ schedule, intermittent ${D}_{1}$ schedules with different on- and off-dosing durations, and adaptive schedules where the use of ${D}_{1}$ and ${D}_{2}$ were guided by ctDNA measurements, were simulated and evaluated to identify optimal treatment designs (Table 2). For adaptive schedules, the treatment started with a continuous ${D}_{1}$ and switched to a continuous ${D}_{2}$ when the ctDNA measurements increased to an upper limit for drug adjustment. When the mutation concentration decreased back to a lower limit for drug adjustment, the treatment was switched back to ${D}_{1}$ and the loop continued.

**Table 2:** Evaluated treatment schedules.

Schedules	Details		
Continuous schedule (standard of care)	${D}_{1}$ was continuously administered resulting in continuous drug exposure for 180 weeks		
Intermittent schedules	${D}_{1}$ was administered for N weeks and suspended for M weeks. Total treatment time was 180 weeks		
	N (weeks)	M (weeks)	
	4	4, 8	
	8	4, 8, 12	
	12	4, 8, 12, 16	
Adaptive schedules with a hypothetical second treatment	${D}_{1}$ was continuously given, and suspended and switched to ${D}_{2}$ when the ctDNA measurement increased to higher than UP fragment/ml. Treatment switched back to ${D}_{1}$ when ctDNA measurement decreased back to lower than LOW fragment/ml. Total treatment time was 180 weeks		
	LOW (fragment/ml)	UP (fragment/ml)	Monitoring frequency of ctDNA (weeks)
	5	10, 15, 20, 25	4
	10	15, 20, 25	4
	5	10, 15, 20, 25	8
	10	15, 20, 25	8
	5	10, 15, 20, 25	12
	10	15, 20, 25	12

${D}_{1}$, anti-EGFR inhibitor; ${D}_{2}$, a hypothetical second treatment to which the newly acquired clone is susceptible; ctDNA, circulating tumor DNA. Drug exposure variability was not considered in this study but only the presence (${D}_{n}$=1) or absence (${D}_{n}$ =0) of a drug were considered.

**Figure 3:**
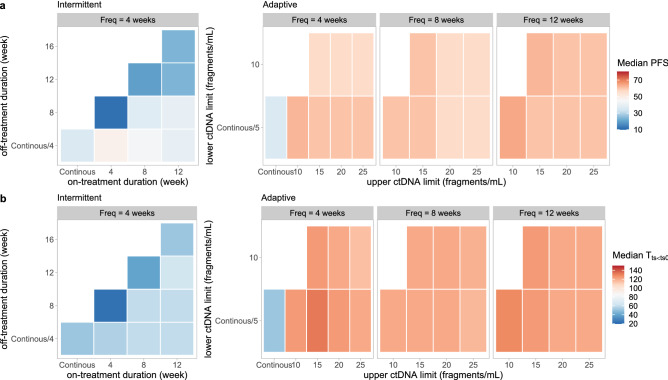
The predicted median progression-free survival (PFS) (**a**) and the time until the tumor size had grown back to the baseline level (T_TS<TS0_) (**b**) of evaluated regimens.

**Figure 4:**
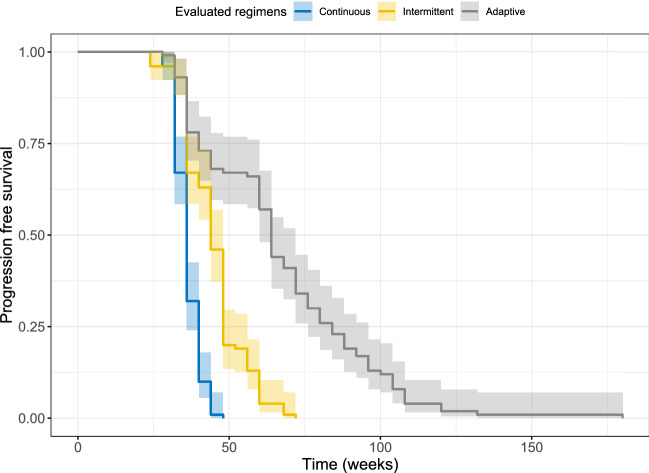
The survival plot of 100 virtual patients under continuous treatment, intermittent treatment (8-week treatment and 4-week suspension), and adaptive treatment with the second hypothetical drug (ctDNA limits for drug adjustment: 5 and 10 fragments/ml, monitor frequency: 12 weeks).

Predicted median progression-free survival (PFS) and time until the tumor size had grown back to the baseline level (T_TS<TS0_) of the simulated population under all evaluated regimens are shown in Fig. 3, the detailed results of which can be found in Supplementary Table S4. The median predicted PFS under continuous drug exposure was 36 weeks and median T_TS<TS0_ was 52 weeks. Five out of 9 designs of intermittent schedule prolonged median PFS and median T_TS<TS0_ compared with continuous treatment (Fig. 3). Four- or 8-week treatment suspension was introduced in these regimens. Extending the treatment holiday from 4 to 4 weeks more than the treatment period mostly resulted in inferior results (Fig. 3). A regimen consisting of 4-week treatment and 4-week suspension (S_interm(4on_4off)_) provided the longest median PFS (48 weeks), while a schedule consisting of 8-week treatment and 4-week suspension (S_interm(8on_4off)_) provided the longest T_TS<TS0_ (60 weeks). A survival prediction also illustrated a better clinical outcome provided by regimen S_interm(8on_4off)_ than continuous regimen (Fig. 4).

As for the adaptive regimen guided by ctDNA measurements, all designs further prolonged median PFS to 56–64 weeks and T_TS<TS0_ to 114–132 weeks (Fig. 3). Comparable results were obtained when the monitoring frequency of ctDNA altered and slightly longer median PFSs were observed when the monitoring frequency of ctDNA was once every 12 weeks. Under the same monitoring frequency, the different upper and lower ctDNA limits for drug adjustment only resulted in small changes in median PFS and T_TS<TS0_, especially when the ctDNA was less frequently monitored. Overall, the longest median PFS and T_TS<TS0_ were mostly observed when the upper and lower ctDNA limits for drug adjustment were 5 fragments/ml and 10 fragments/ml, respectively (Fig. 3). A regimen with 5 and 10 fragments/ml as ctDNA limits for drug adjustment and a monitoring frequency of once every 12 weeks (S_adapt(5_10_Freq12)_) provided the longest median PFS. The survival prediction of S_adapt(5_10_Freq12)_ also showed a better clinical outcome than the regimen S_interm(8on_4off)_ and the continuous regimen (Fig. 4).

**Figure 5:**
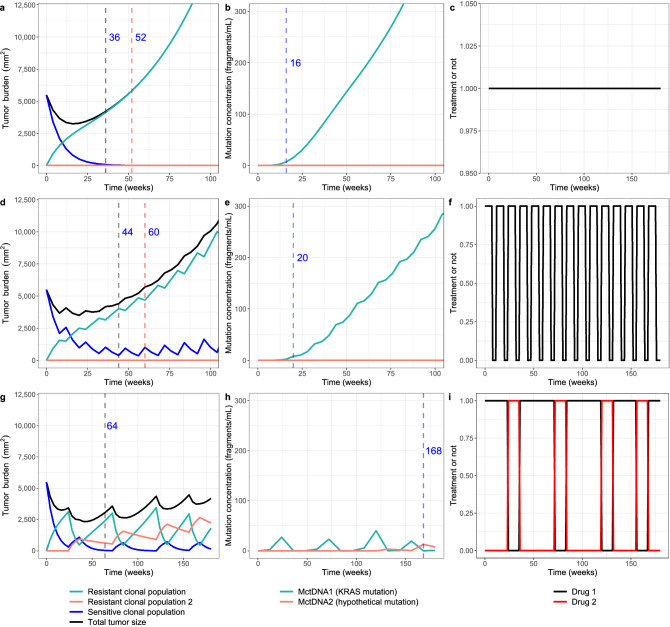
The simulated time-curves of total tumor burden and each clonal population (**a**, **d**, **g**), mutation concentrations (**b**, **e**, **h**), and dosing strategies (**c**, **f**, **i**) of a typical subject with metastatic colorectal cancer undergoing continuous treatment (**a**, **b**, **c**), intermittent treatment (8-week treatment and 4-week suspension) (**d**, **e**, **f**), and adaptive treatment with the second hypothetical drug (ctDNA limits for drug adjustment: 5 and 10 fragments/ml, monitor frequency: 12 weeks) (**g**, **h**, **i**). Estimated PFS (black dashed vertical line), T_TS<TS0_ (red dashed vertical line), and T_mutant_test_ (blue dash vertical line) are also shown in the figure.

Figure 5 shows the simulated time-curves of each tumor clonal population and each mutation in ctDNA over time from a typical subject under the continuous schedule, the intermittent schedule S_interm(8on_4off)_, and the adaptive schedule S_adapt(5_10_Freq12)_. The corresponding results of the simulated population are shown in Supplementary Fig. S3. It can be seen that the schedule S_interm(8on_4off)_ and S_adapt(5_10_Freq12)_ suppressed the growth of resistant clonal population ${T}_{R1}$. In addition, predicted time until detectable mutation (T_mutant_test_) under each evaluated regimen was evaluated. It was shown that ${M}_{ctDNA1}$ under both continuous and intermittent regimens could become detectable before disease progression (Fig. 5, Table S4). In the setting of adaptive treatment, as the ${M}_{ctDNA1}$ level was applied as a biomarker to guide the treatment switching, the median T_mutant_test_ of ${M}_{ctDNA2}$ was evaluated. The results indicate that ${M}_{ctDNA2}$ would be observed after disease progression has occurred but before the tumor size grows back to baseline level (Fig. 5).

### Sensitivity analysis

While the value of the parameters describing tumor dynamics were estimated based on the data or adapted from literature, that of other parameters were set based on a visual fit to the data since the amount of data did not support estimation of parameters. These parameter values may however not be optimal, and therefore the parameter sensitivity to the simulated curves was assessed by increasing or decreasing parameters by 50% one at a time.

**Figure 6:**
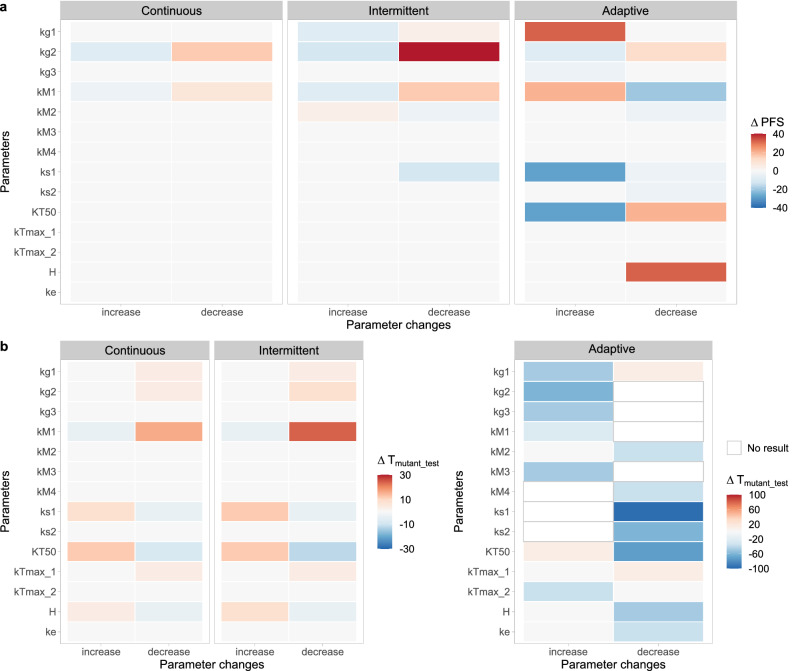
Relative change (Δ) of predicted progression-free-survival (weeks) (**a**) and time until detectable mutation (weeks) (**b**) compared with using original parameters in the sensitivity analysis. No result, the mutant gene concentrations did not reach the detectable limit (5 fragments/ml) by the end of simulation time (180 weeks).

The predicted PFS and T_mutant_test_ derived from each time of simulation, which represent the dynamics of tumor burden and mutation concentrations in ctDNA respectively, are shown in Fig. 6 and Supplementary Table S5. Both simulated tumor sizes and mutation concentrations were affected when any of the parameters characterizing the tumor burden dynamics, including net growth rate constants (${k}_{g}$), tumor shrinkage rate due to treatments (${k}_{s}$), and mutation rate constants (${k}_{M}$) varied. In contrast, the change of the parameters characterizing the mutation concentrations, including the Hill coefficient ($H$), max releasing rates (${k}_{max}$), the tumor size that provide half-maximal releasing rate (${KT}_{50}$), and elimination rate constant of ctDNA (${k}_{e}$), only affected the simulated mutation concentrations but not the simulated tumor size except for ${KT}_{50}$ and $H$ under an adaptive treatment design. The predicted PFS was mainly sensitive to parameters ${k}_{g2}$ and ${k}_{M1}$, and the predicted T_mutant_test_ was mainly sensitive to parameters ${k}_{s1}$, ${k}_{M1}$, $H$ and ${KT}_{50}$. Nonetheless, the intermittent regimen and the adaptive regimen still resulted in better treatment outcomes (i.e. longer PFS) than the continuous regimen, no matter how the parameter values varied (Table S5). More detailed simulated time-curves of tumor burden and ${M}_{ctDNA1}$ concentrations under each setting, and the relative changes of predicted total tumor sizes and ${M}_{ctDNA}$ levels compared with original results are shown in Supplementary Fig. S4 and Fig. S5.

## Discussion

In the current study, a mathematical model was developed to characterize the tumor size dynamics and tumor resistance development in response to treatment. The model was built based on findings from previously published studies and the collected raw data itself. The model well captured the reported time curves of tumor sizes and mutant *KRAS* levels in ctDNA from mCRC patients. A similar model could also characterize the time-curves of EGFR mutation and tumor sizes obtained from NSCLC patients.

The current model assumed that for patients who had no detectable *KRAS* mutation pre-treatment, there was no primary resistance, despite that the original study estimated that drug resistance is likely to be present prior to the initiation of treatment^13^. However, since the size of the resistant clonal population was estimated to only account for a small part of the total tumor cell population (2300 cells out of one billion cells)^13^, the primary resistance was eventually not included in our model.

During treatment interruption, a back transfer process from drug resistant clonal population to drug sensitive clonal population was incorporated to capture the recovery of sensitivity to the treatment. This assumption was supported by in vitro observations^9^. This process could also describe the phenomenon that in the absence of the drug, susceptible tumor cells have the benefit to grow back again at the expense of resistant tumor cells. When the back transfer process was removed (${k}_{M2}$ and ${k}_{M4}$ fixed to 0), prolonged predicted median PFSs under the schedule S_interm(4on_4off)_ and S_adapt(5_10_Freq12)_ compared with the continuous regimen were still observed, although not for schedule S_interm(8on_4off)_ in contrast to when the back transfer was allowed (Supplementary Fig. S6, Fig. S7). However, the decline of ctDNA upon withdrawal of treatment, which has been observed in mCRC patients^9,34^, could not be captured when removing the back transfer process (Supplementary Fig. S8). It was also observed that under this circumstance, the remaining susceptible cells had no growth advantage over the resistant cells during the withdrawal of treatment, hence tumor would not regain susceptibility (Supplementary Fig. S7, Fig. S8). Therefore, the back transfer process is considered to be a reasonable assumption to describe the dynamics of and the competition among different clonal populations upon treatment withdrawal based on current available data. More data under intermittent therapy would be valuable to better characterize this dynamic process, and to better estimate parameters.

A delayed emergence of a mutation indicating treatment resistance in ctDNA was observed in both original studies on mCRC patients (after in median 22 weeks’ treatment)^13^ and NSCLC patients (after in median 10.5 months’ treatment)^14^. This phenomenon was characterized by the Hill equations with tumor size as the independent variable (Eq. (4) and (5)) in the current study, assuming a delayed shedding of ctDNA from the tumor tissue. We also investigated a model where the delayed process was incorporated in the mutation from one clonal population to another by applying transit compartments. This model could also capture the delayed emergence of mutation in ctDNA.

The designs of intermittent and adaptive regimens aim to prolong the duration of suppressing treatment resistance since they considered intra-tumor heterogeneity and evolving adaptation of tumor to treatment. In addition, the evaluated adaptive schedules also enabled personalized design of therapy since the switch of drug was guided by individual ctDNA measurements. Here we focused explicitly on the use of ctDNA and therefore the change in tumor size was not considered as a criterium to switch therapy, despite the fact that tumor size is a common marker in clinical practice for the efficacy of anti-cancer treatment^11^. In the future, the help of tumor size could be further evaluated when data regarding ctDNA and tumor size dynamics under adaptive therapy are available to facilitate better understanding of their relationship and refining the current model.

In the current study, the intermittent and adaptive regimens, with appropriate designs, were shown to outperform the conventional continuous treatment by simulations (i.e. median PFS was prolonged) (Fig. 3). This is in line with the evolutionary principle of control and the findings from clinical observations. For example, an adaptive intermittent treatment of abiraterone based on prostate-specific antigen (PSA) levels was shown to result in a better clinical outcome than the typical continuous treatment^19^, although the study design may need to be refined^35^. Another recent retrospective analysis demonstrated that intermittent use of enzalutamide in metastatic castration-resistant prostate cancer patients prolonged the time to PSA failure and improved overall survival^20^. Traditional approaches to cancer therapy have not exploited these theoretical advantages. For example, current protocols typically apply a treatment agent or agents at the maximum tolerated dose (MTD) until there is unequivocal clinical evidence of progression^21^.

The intermittent therapy has also been investigated in several clinical studies. In contrast to our simulation results and the clinical observations, these studies did not show improved outcome in patients undergoing intermittent therapy^22–27^. One study on BRAF and MET inhibitors in melanoma patients even showed an inferior result under the intermittent therapy compared to continuous therapy^22^. The underlined mechanism remains unclear. Nevertheless, in these cases, the developed mathematical model may be helpful for understanding these conflicting results. Further identification of optimal designs based on different resistance mechanism and dynamics of cancers can be supported by the model-based approach. For example, a previous in silico study showed that an intermittent abiraterone followed by a lead-in period was not beneficial for prostate cancer patients, and the adaptive intermittent treatment guided by PSA was demonstrated to be the best option^19^. Moreover, the simulation results derived from the current study suggest that although introducing a treatment holiday may improve the treatment outcome, the length of treatment holiday still needs to be controlled. Extending the treatment holiday mostly resulted in inferior results, especially when the holiday was longer than the treatment period. This is in accordance to a previous finding that chemotherapy with shorter intervals (dose-dense therapy) resulted in better treatment outcome even though the total dose amounts were same^36^.

When evaluating the adaptive treatment, a second hypothetical treatment (${D}_{2}$) targeting ${T}_{R1}$ was introduced. An example of this idea can be seen from the treatments of NSCLC patients. For NSCLC patients, acquisition of T790M mutation is the main mechanism of acquired resistance upon treatment of erlotinib/gefitinib, and osimertinib can be selected for T790M-positive patients^37^. Lately, the Food and Drug Administration (FDA) also granted accelerated approval to the first KRAS-blocking drug^38^. This indicates a potential feasibility of the here suggested adaptive treatment design. Due to the use of ${D}_{2}$, a hypothetical newly acquired mutation (${M}_{ctDNA2}$) was also considered in the model. Unlike ${M}_{ctDNA1}$ (*KRAS* mutation), ${M}_{ctDNA2}$ only became detectable after disease progression in the current study. This brings on a question about the predictive value of mutations in ctDNA. Most likely the dynamics of the sensitive clones are also very important to predict emerging resistance at an earlier phase. However, to answer this question, more data is required to support the understanding of the dynamics of the hypothetical mutation.

With the sensitivity analysis we showed that the choice of parameter values can affect the simulated curves. The predicted tumor sizes were mainly sensitive to the parameters ${k}_{g2}$ and ${k}_{M1}$ using the developed model, and the predicted mutation concentrations were mainly sensitive to the parameters ${k}_{s1}$, ${k}_{M1}$, $H$ and ${KT}_{50}$ (Fig. 6). This suggests that an accurate estimation of these parameters is of importance for this model. However, the intermittent and adaptive treatment still provided better treatment outcome when parameter values varied, indicating that the value of the parameters didn’t affect the conclusion that the intermittent and adaptive regimens with a certain design outperform the conventional continuous treatment.

To apply the novel treatment strategy, there are still some challenges. Firstly, for patients who had detectable *KRAS* mutation pre-treatment, the intermittent treatment provided similar treatment outcome compared to continuous treatment (Supplementary Fig. S9). Therefore, for these patients, a better option will be to choose another treatment from start. In fact, in clinical practice panitumumab is contraindicated for patients with *KRAS* mutation. Secondly, to be able to monitor the development of resistance with ctDNA, the mutations that are associated with the resistance to a target treatment need to be acknowledged beforehand. If multiple mutations have been reported, a selection may be required based on the capability of the applied quantification technique, such as the selection of gene panel in the assay and the number of mutations that can be detected simultaneously. Thirdly, as can be seen from the previous study, only 9 out of 25 patients developed detectable *KRAS* mutations and the median disease progression time of the 9 patients was same as for the remaining 16 patients (23 weeks). It was also noticed when the individual results were compared, 4 out of 100 virtual patients were predicted to have longer PFS under a continuous schedule than under regimen S_interm(8on_4off)_. Additionally, despite that adaptive regimens provided longer median PFS than intermittent regimens, 31 out of 100 patients had longer PFS under regimen S_interm(8on_4off)_ than under regimen S_adapt(5_10_Freq12)._ These results indicate that ctDNA guided treatment may not be feasible for all patients and variability between individuals can affect the choice of regimen.

Our study has some limitations. First of all, the amount of data we obtained limited the ability to adequately estimate all parameters of the developed model. We were also not able to fully consider pre-treatment tumor heterogeneity and incorporate the eco-evolutionary dynamics in the model. Additionally, due to the lack of drug exposure records, dose- or exposure–response relationship was not incorporated in the model and was not investigated in this study. However, for panitumumab, it has been shown that with standard treatment regimens, even the trough concentrations are maintained above the 90% saturation levels, meaning almost maximum effect in all patients^39^. However, for other molecules such as tyrosine kinase inhibitors (TKIs), drug levels are also important to be included in the analysis. In these cases, drug exposure measurements can be helpful for the understanding of exposure–response relationship under the evaluated regimens. Secondly, alternative mutations that are related to anti-EGFR treatment resistance in addition to the reported mutant genes were not considered in this study. However, *KRAS* mutation and *EGFR* mutation were the most commonly reported gene mutations that are associated with resistance to anti-EGFR treatment in mCRC and NSCLC patients respectively^18^. Therefore, we mainly considered the most representative mutations. Thirdly, the idea of individual intermittent treatment could be further investigated. Because of the above limitations, an external dataset is needed to validate the results and a clinical pilot study is required to confirm the added value of the suggested schedules.

In conclusion, a mathematical model incorporating evolving cancer resistance was developed to characterize tumor size dynamics and resistance development under treatment. The model well captured the clinical data from colorectal cancer patients as well as from NSCLC patients. Compared with a conventional continuous anti-cancer treatment schedule, intermittent and adaptive schedules were predicted to better suppress the evolving cancer resistance and suggested a potential improvement of clinical outcome. However, a prospective study is required to validate the results and to confirm the added value of the suggested approach.

## Methods

### Dataset

A dataset containing longitudinal tumor burden measurements and mutant *KRAS* levels in ctDNA was identified from a published study where patients diagnosed with mCRC were treated with the anti-EGFR inhibitor panitumumab^13^. Patient demographic information, time-courses of tumor burden that was reported as the aggregate cross-sectional diameter of all index lesions (mm^2^), and the time-courses of mutant *KRAS* concentrations (fragments/ml) of 28 patients were collected from the supplementary tables of the paper^13^. When corresponding time of a data point was not shown in the table, the time information was digitized from the corresponding supplementary figures using WebplotDigitizer (https://apps.automeris.io/wpd/).

All data in this study were collected from publicly available materials (i.e. supplementary material or figures) in literature from which the studies were approved by corresponding ethical committees and all informed consents were obtained. Therefore, for this study, no additional ethical approval or written informed consent was required. All procedures in this study were performed in accordance with relevant guidelines.

### Model structure

A mathematical model was developed to describe the obtained time-courses of tumor burden and mutant *KRAS* concentrations under anti-EGFR therapy. The model structure is shown in Fig. 2.

Six assumptions were made when developing the model structure:

1. The growth of the tumor was assumed to follow an exponential growth pattern^40,41^.
2. Tumor tissue was assumed to consist of multiple clonal sub populations which are defined as sets of cancer cells that share a common genotype^5^. One clonal population ($${T}_{s}$$) was defined to be sensitive to the anti-EGFR inhibitor panitumumab ($${D}_{1})$$. Another clonal population ($${T}_{R1})$$ harbored *KRAS* mutation ($${M}_{ctDNA1}$$) and was consequently resistant to $${D}_{1}$$. This is based on previous evidence where patients harboring *RAS* variant in pre-treatment ctDNA did not benefit from EGFR blockade^13,42^. The emergence of *KRAS* mutation was also suggested to be a mediator of acquired resistance to EGFR blockade^13,42^.
  For patients who were initially identified as *KRAS* wild-type in ctDNA (WT-*KRAS* patients), ${T}_{s}$ was assumed to form the whole tumor at the start of treatment. While for patients who had detectable mutant *KRAS* in ctDNA pre-treatment (M-*KRAS* patients), tumor tissue was assumed to consist of both ${T}_{s}$ and ${T}_{R1}$ at the start of treatment. In addition, given that the resistant clonal population may have fitness cost^43^, the proliferation rate of resistant clones was assumed to be lower than that of the sensitive clones^44^.
3. A *KRAS* mutation could be acquired during the treatment of ${D}_{1}$, as WT-*KRAS* patients could develop detectable mutations^13^.
4. A hypothetical treatment next to panitumumab ($${D}_{2})$$ was incorporated in the current study and assumed to target *KRAS*-mutated colorectal cancer and thereby inhibiting the growth of $${T}_{R1}$$. In the meantime, a second mutation ($${M}_{ctDNA2}$$) was able to be acquired which resulted in a third clonal population ($${T}_{R2}$$) that was resistant to $${D}_{2}$$. The mutation rate was assumed to be the same as that of the acquiring *KRAS* mutation clonal population.
5. During treatment interruption, a back transfer process from the drug resistant clonal population to drug sensitive clonal population was assumed to be present and was incorporated in the model with a rate lower than the mutation rate. This assumption was supported by a previous in vitro study in colorectal cancer (CRC) cells^9^, which showed that CRC cells that acquired resistance to cetuximab with amplification of *KRAS* gene regained partial sensitivity to cetuximab when cultured in the absence of the drug^9^. This process could also be understood as the competition between drug susceptible and resistant cells in the absence of the drug. When the pressure of the drug was gone, the susceptible cells have the benefit to grow back again at the expense of resistant cells in the tumor.
6. ctDNA which carries the target mutations was shed from resistant clonal populations and the shedding rate depends on the corresponding tumor tissue size.

In order to be able to capture the following features observed from clinical studies, two features were incorporated in the model structure:

1. The mutant *KRAS* concentration became detectable after 5–34 weeks’ (median 22 weeks) treatment for WT-*KRAS* patients who developed detectable mutant *KRAS*^13^. Therefore, the Hill equations (Eq. (4) and (5)) were applied to describe this delayed emergence (or ability to detect) of ${M}_{ctDNA1}$ and ${M}_{ctDNA2}$.
2. Mutant *KRAS* levels in ctDNA increased when challenged with ${D}_{1}$ and declined upon the withdrawal of treatment^9^. The elimination half-life of resistance mutations is approximately 4 months^34,42^. Therefore, in addition to the back transfer process, a first-order ctDNA elimination was incorporated. The half-life of a typical patient was confirmed to be 4.15 months with the given parameter values.

The ordinary differential equations of the model were as follows:

1 $$\frac{d{T}_{s}}{dt}={k}_{g1}\cdot {T}_{s}-{k}_{s1}\cdot {D}_{1}\cdot {T}_{s}-{k}_{M1}\cdot {D}_{1}\cdot {T}_{s}+{k}_{M2}\cdot {(1-D}_{1})\cdot {T}_{R1}$$

2 $$\frac{d{T}_{R1}}{dt}={k}_{M1}\cdot {D}_{1}\cdot {T}_{s}+{k}_{g2}\cdot {T}_{R1}-{k}_{s2}\cdot {D}_{2}\cdot {T}_{R1}-{k}_{M2}\cdot {(1-D}_{1})\cdot {T}_{R1}-{k}_{M3}\cdot {D}_{2}\cdot {T}_{R1}+{k}_{M4}\cdot (1-{D}_{2})\cdot {T}_{R2}$$

3 $$\frac{d{T}_{R2}}{dt}={k}_{M3}\cdot {D}_{2}\cdot {T}_{R1}+{k}_{g3}\cdot {T}_{R2}-{k}_{M4}\cdot (1-{D}_{2})\cdot {T}_{R2}$$

4 $${k}_{1}={k}_{\mathrm{max}\_1}\cdot {{T}_{R1}}^{H}/ ({{T}_{R1}}^{H}+{{KT}_{50}}^{H})$$

5 $${k}_{2}={k}_{\mathrm{max}\_2}\cdot {{T}_{R2}}^{H}/ ({{T}_{R2}}^{H}+{{KT}_{50}}^{H})$$

6 $$\frac{d{M}_{ctDNA1}}{dt}={k}_{1}\cdot {T}_{R1}-{k}_{e}\cdot {M}_{ctDNA1}$$

7 $$\frac{d{M}_{ctDNA2}}{dt}={k}_{2}\cdot {T}_{R2}-{k}_{e}\cdot {M}_{ctDNA2}$$

8 $$TS={T}_{s}+{T}_{R1}+{T}_{R2}$$

$TS$ represents the total tumor size as detected by CT scan. ${k}_{g1}$, ${k}_{g2}$, and ${k}_{g3}$ represent the net growth rate constants of three clonal populations. ${k}_{s1}$ and ${k}_{s2}$ represent the tumor shrinkage rate due to treatments. Drug exposure variability was not considered in this study but only the presence (${D}_{n}$=1) or absence (${D}_{n}$=0) of a drug were considered ($n$=1 and 2 represent panitumumab and the hypothetical treatment, respectively). ${k}_{M1}$ and ${k}_{M3}$ represent the mutation rate constants governing the transfer from the drug susceptible clonal population to the drug resistant clonal population during ${D}_{1}$ and ${D}_{2}$ treatment, respectively. ${k}_{M2}$ and ${k}_{M4}$ represent the mutation rate constants from drug resistant clonal population to drug susceptible clonal population upon the withdrawal of treatments. ${k}_{1}$ and ${k}_{2}$ represent the shedding rate constants of ctDNA which carries mutations. Hill equations (Eq. (4) and (5)) was applied to capture the concentration change of ${M}_{ctDNA}$. ${k}_{\mathrm{max}\_1}$ and ${k}_{\mathrm{max}\_2}$ are max releasing rates, ${KT}_{50}$ is the tumor size that provide half-maximal releasing rate, ${H}$ is the Hill coefficient.${k}_{e}$ represent the elimination rate constant of ctDNA.

When performing simulations, the baseline levels of $TS$ (Eq. (8)) and ${M}_{ctDNA1}$ were set to the median of the real observations in different patient groups (Supplementary Table S1). For WT-*KRAS* patients, the baseline ${T}_{R1}$ (${T}_{R1\_0}$) and ${T}_{R2}$ (${T}_{R2\_0}$) were both set to 0. For M-*KRAS* patients, ${T}_{R2\_0}$ were set to 0 while ${T}_{R1\_0}$ was set according to the median of observations.

### Parameter values

The values of all model parameters used in the simulation are shown in Table 1.

To assist the setting of parameter values, the parameters describing tumor dynamics under ${D}_{1}$ therapy (${k}_{s1}$ and ${k}_{M1}$) were estimated by fitting the collected tumor sizes data using the first order conditional estimation method with interaction (FOCEI) implemented in the NONMEM software, version 7.4.1 (ICON Development Solutions). The detailed method on parameter estimates can be found from the Supplementary methods.

The estimated typical values of ${k}_{s1}$ and ${k}_{M1}$ were adopted to simulations. Assuming the tumor growth follows an exponential growth pattern, ${k}_{g1}$ was fixed as 0.03/week (= ln2/(6.8 months * 4 weeks/month)) according to a previously reported median placebo tumor doubling time of colorectal carcinomas, i.e. 6.8 months (range: 3–24 months)^40^. Accordingly, ${k}_{g2}$ was fixed as 0.021/week (0.03 * 70%). ${k}_{M2}$ was set to be lower than ${k}_{M1}$ based on the 5th assumption. The parameters that are related to the emergence of mutations ($H$, ${KT}_{50}$, and ${k}_{max}$) were set by visually matching the slope of mutant *KRAS* time-courses and the detectable time of mutant *KRAS*.

Random IIV was incorporated on ${k}_{g}$ and baselines, which was assumed to be log-normally distributed, when performing the simulations (Table 1). It was due to the fact that patients in the dataset had different baseline tumor burden and mutant *KRAS* levels, and different growth rates of CRC were reported in different studies^13,40^. If data from more patients can be included, the IIV on parameters will be able to be added on more parameters and be estimated.

### Model evaluation

To evaluate the suitability of the model, five hundred times of simulation were performed for $TS$ and ${M}_{ctDNA1}$ concentrations under continuous drug exposure. The 50^th^ percentiles and the corresponding 95% CIs of simulations derived from the model were plotted along with the real observation points and the 50^th^ percentiles of observations. In addition, assuming ${D}_{1}$ was administered continuously for 20 weeks (leading to a continuous drug exposure) and then stopped for 20 weeks, the time-course of ${M}_{ctDNA1}$ concentrations were simulated for 100 virtual patients to demonstrate if the decay upon the withdrawal of treatment could be captured by this model.

The performance of the model was also evaluated using another dataset from a study on NSCLC patients receiving EGFR inhibitors (icotinib/gefitinib) with the same method as above^14^. The time curves of tumor size which was reported as the longest diameter (mm) and that of EGFR mutation (L858R, exon 19 deletion and T790M) concentrations (mutation copies/ml plasma) detected from ctDNA were digitized from published figures using WebplotDigitizer (https://apps.automeris.io/wpd/). The model used in the evaluation cohort was adjusted according to the findings of the study. More detailed introduction of the model and parameter values is shown in Supplementary methods.

### Treatment schedule evaluation

Treatment schedules that were considered in the current study are shown in Table 2. These schedules were evaluated on WT-*KRAS* patients.

A continuous schedule with ${D}_{1}$ was first considered. The continuous schedule is the conventional treatment strategy in clinical practice where a therapy is administered continuously until disease progression (i.e. in schedules leading to continuous drug exposure)^19^. Monitoring frequency, i.e. the frequency of taking a blood samples for ctDNA analysis and assessing tumor sizes, was set as once every 4 weeks according to the frequency of the obtained data.

To identify an optimized anti-cancer treatment schedules that suppresses the development of treatment resistance, intermittent schedules with ${D}_{1}$ and adaptive schedules with ${D}_{1}$ and ${D}_{2}$ guided by ctDNA measurements, as proposed in previous studies^2,8,19,21^, were considered. For the intermittent schedules, drug-exposure interruption was introduced and multiple combinations of on- and off-dosing durations were evaluated. For the adaptive schedules, the ctDNA measurements were monitored and applied as a biomarker to determine the time point of switching treatment between ${D}_{1}$ and ${D}_{2}$. The treatment started with ${D}_{1}$ and continued till the ctDNA measurements increased to an upper limit for drug adjustment. Then ${D}_{1}$ was suspended and switched to a continuous ${D}_{2}$. When the mutation concentration decreased back to a lower limit for drug adjustment, the treatment was switched back to ${D}_{1}$ and the loop continued. In this case, multiple monitoring frequencies of ctDNA and multiple threshold of mutation concentrations for treatment switching were explored for comparison. The frequency of assessing tumor sizes was set as once every 4 weeks.

Simulations were performed with the package RxODE (version 1.0.8) implemented in R (version 4.0.2). One hundred virtual patients were simulated under each regimen. PFS of each virtual patient under each schedule was derived from the simulated total tumor size at every monitoring time point. PFS was defined based on WHO criteria (i.e. 25% increase in $TS$) as was applied in the original study^13,45^. The T_TS<TS0_ was also estimated to compare the effect of different regimens. In addition, T_mutant_test_ was estimated assuming a lower limit of quantification for target mutant genes in ctDNA of 5 fragments/ml which was set based on the observed data. This aimed to determine if detectable mutation in ctDNA can be a predictor of disease progression.

### Sensitivity analysis

A sensitivity analysis was performed to evaluate the impact of all parameter values on the model predictions. Every parameter was set as 50% or 150% of the original typical values one at a time. The continuous schedule, one intermittent schedule S_interm(8on_4off)_, and one adaptive schedule S_adapt(5_10_Freq12)_ were simulated. IIV was not incorporated here. The sensitivity to the parameters was assessed by comparing the newly simulated time-courses of total tumor size and mutation concentrations with the original simulations results. Median PFS and T_mutant_test_ derived from each simulation was also estimated for comparison.

## Supplementary Information

Supplementary Information.

## Data Availability

The datasets generated during and/or analyzed during the current study are available from the corresponding author upon reasonable request.
