# Publisher Correction: Anti-cancer treatment schedule optimization based on tumor dynamics modelling incorporating evolving resistance

**DOI:** 10.1038/s41598-022-09014-1

**Published:** 2022-03-22

**Authors:** Anyue Yin, Johan G. C. van Hasselt, Henk-Jan Guchelaar, Lena E. Friberg, Dirk Jan A. R. Moes

**Affiliations:** 1 Department of Clinical Pharmacy and Toxicology, Leiden University Medical Center, Albinusdreef 2, 2333 ZA Leiden, The Netherlands; 2 Leiden Network for Personalized Therapeutics, Leiden University Medical Center, Leiden, The Netherlands; 3 Division of Systems Biomedicine and Pharmacology, Leiden Academic Centre for Drug Research (LACDR), Leiden University, Leiden, The Netherlands; 4 Department of Pharmacy, Uppsala University, Uppsala, Sweden

Correction to: *Scientific Reports* 10.1038/s41598-022-08012-7, published online 10 March 2022

The original version of this Article contained errors in Table 1 where the data was listed incorrectly in the column ‘M-*KRAS* patients’. The original Table 1 and accompanying legend appear below.

**Table 1:** Parameters values of the developed model characterizing the dynamics of tumor size and mutation concentrations in metastatic colorectal cancer (mCRC) patients.

Parameters (units)	Description	Typical values		Reference
		WT-*KRAS* patients	M-*KRAS* patients	
$T_{s\_0}$(mm^2^)	Baseline of $T_{s}$ (clonal population that is sensitive to anti-EGFR inhibitor ($D_{1}$) )	5500	100	Data/Estimated value; Mutation was assumed to be acquired during treatment
$T_{R1\_0}$(mm^2^)	Baseline of $T_{R1}$ (clonal population that is resistance to $D_{1}$ but is sensitive to the second hypothetical treatment ($D_{2}$))	0	1700	Data/Estimated value; Mutation was assumed to be acquired during treatment
$T_{R2\_0}$(mm^2^)	Baseline of $T_{R2}$ (clonal population that is resistance to both treatments)	0	0	Data/Estimated value; Mutation was assumed to be acquired during treatment
$M_{ctDNA1\_0}$(fragments/ml)	Baseline of mutant *KRAS* $\left( {M_{ctDNA1} } \right)$ in ctDNA	0	500	Data/Estimated value; Mutation was assumed to be acquired during treatment
$M_{ctDNA2\_0}$(fragments/ml)	Baseline of a second hypothetical mutation $\left( {M_{ctDNA2} } \right)$ in ctDNA	0	0	Data/Estimated value; Mutation was assumed to be acquired during treatment
$k_{g1}$(/week)	Growth rate constant of $T_{s}$	0.03		^40^
$k_{g2}$(/week)	Growth rate constant of $T_{R1}$	0.021		^43,44^
$k_{g3}$(/week)	Growth rate constant of $T_{R2}$	0.015		^43,44^
$k_{s1}$(/week)	Tumor shrinkage rate constant due to $D_{1}$	0.1		Estimated value
$k_{s2}$(/week)	Tumor shrinkage rate constant due to $D_{2}$	0.1		$k_{s1}$
$k_{M1}$(/week)	Mutation rate from $T_{s}$ to $T_{R1}$ when $D_{1}$ = 1	0.05		Estimated value
$k_{M2}$(/week)	Mutation rate from $T_{R1}$ to $T_{s}$ when $D_{1}$ = 0	0.03		Lower than $k_{M1}$ ^9^
$k_{M3}$(/week)	Mutation rate from $T_{R1}$ to $T_{R2}$ when $D_{2}$ = 1	0.05		$k_{M1}$
$k_{M4}$(/week)	Mutation rate from $T_{R2}$ to $T_{R1}$ when $D_{2}$ = 0	0.03		$k_{M2}$
$H$	Hill coefficient	5		Visually matching the slope of data and the detectable time of mutant *KRAS*
$KT_{50}$(mm^2^)	The size of tumor that provide half-maximal shedding rate of ctDNA	3500		Visually matching the slope of data and the detectable time of mutant *KRAS*
$k_{{{\text{max}}\_1}}$((fragments/ml)/(week*mm^2^))	Maximum shedding rate of $M_{ctDNA1}$	0.015	1.5	Visually matching the slope of data and the detectable time of mutant *KRAS*
$k_{e}$(/week)	ctDNA eliminate rate constant	0.5		Visually matching the slope of data and the detectable time of mutant *KRAS*
$k_{{{\text{max}}\_2}}$((fragments/ml)/(week*mm^2^))	Maximum shedding rate of $M_{ctDNA2}$	0.015	1.5	$k_{{{\text{max}}\_1}}$
IIV_ $B$ ($\omega_{1}$)	Standard deviation of IIV of baselines	0.6		Data
IIV_ $k_{g}$ ($\omega_{2}$)	Standard deviation of IIV of $k_{g}$	0.2		Data

ctDNA, circulating tumor DNA; IIV, interindividual variability; WT-KRAS patients, patients who were initially identifed as *KRAS* wild-type in ctDNA; M-KRAS patients, patients who had detectable mutant *KRAS* in ctDNA pre-treatment.

The original Article has been corrected.

