# Supplementary material for: Anti-cancer treatment schedule optimization based on tumor dynamics modelling incorporating evolving resistance
Source: Sci Rep. 2022 Mar 10;12:4206. doi: 10.1038/s41598-022-08012-7 (PMC8913638; doi:10.1038/s41598-022-08012-7)
Supplement: Supplementary file 1 — Supplementary Information. [file 41598_2022_8012_MOESM1_ESM.pdf]

Anti-cancer treatment schedule optimization based on tumor dynamics modelling incorporating evolving resistance

Anyue Yin[1,2], Johan G.C. van Hasselt[3], Henk-Jan Guchelaar[1,2], Lena E. Friberg[4*], Dirk Jan A.R. Moes[1,2*]

* these authors shared the senior authorship

1. Department of Clinical Pharmacy and Toxicology, Leiden University Medical Center, Leiden, the Netherlands

2. Leiden Network for Personalized Therapeutics, Leiden University Medical Center, Leiden, the Netherlands

3. Division of Systems Biomedicine and Pharmacology, Leiden Academic Centre for Drug Research (LACDR), Leiden University, Leiden, The Netherlands,

4. Department of Pharmacy, Uppsala University, Uppsala, Sweden.

Corresponding author:

Dr. Dirk Jan A.R. Moes, Email: D.J.A.R.Moes@lumc.nl, Address: Albinusdreef 2, 2333 ZA, Leiden, The Netherlands

Supplementary materials

Contents

Supplementary methods 4

1. Parameter estimate 4

2. Model in a evaluation cohort 4

References 5

Figure S1 The model structure that characterize the dynamics of tumor size and mutation concentrations in ctDNA from NSCLC patients. 6

Figure S2. Model evaluation results on the time-courses of tumor diameters (a) and *EGFR* mutation concentrations including L858R mutation/ exon 19 deletion (b) and T790M mutation (c) collected from a previous clinical study where patients with non-small cell lung cancer were treated with anti-EGFR inhibitor icotinib/gefitinib. 7

Figure S3. The simulated total tumor burden (a,c,e) and mutation concentrations (b,d,f) under continuous treatment (a,b), intermittent treatment (8-week treatment and 4-week suspension) (c,d), and adaptive treatment with the second hypothetical drug (ctDNA limits for drug adjustment: 5 and 10 fragments/ml, monitor frequency: 12 weeks) (e.f) for 100 colorectal cancer patients. 8

Figure S4. Simulated time curves from sensitivity analysis. 9

Figure S5. Relative change (Δ) of predicted minimum total tumor size (a), of total tumor size at the last simulated time point (180 weeks) (b), and of M_{ctDNA1} or M_{ctDNA2} concentrations at the last simulated time point (180 weeks) (c) compared with using original parameters in the sensitivity analysis. 10

Figure S6. When fixing k_{M2} and k_{M4} to zero, the predicted median progression-free survival (PFS) (a) and the time until the tumor size had grown back to the baseline level ($T_{TS<TS0}$) (b) of evaluated regimens 11

Figure S7. When fixing k_{M2} and k_{M4} to zero, the simulated time-curves of total tumor burden and each clonal population (a,d,g), mutation concentrations (b,e,h), and dosing strategies (c,f,i) of a typical subject with metastatic colorectal cancer undergoing continuous treatment (a,b,c), intermittent treatment (8-week treatment and 4-week suspension) (d,e,f), and adaptive treatment with the second hypothetical drug (ctDNA limits for drug adjustment: 5 and 10 fragments/ml, monitor frequency: 12 weeks) (g,h,i) 12

Figure S8. When fixing k_{M2} and k_{M4} to zero, model predicted total tumor burden and each clonal population (a) and mutant *KRAS* concentrations (b) under a regimen of 20-week treatment and 20-week suspension. 13

Figure S9 The simulated total tumor burden under continuous treatment (a) and intermittent treatment (8-week treatment and 4-week suspension) (b) for 100 colorectal cancer patients with detectable *KRAS* mutation pre-treatment. 14

Table S1 Characteristics of the dataset collected from patients with metastatic colorectal cancer... 15

Table S2 Parameter estimates of the tumor dynamics model based on the dataset collected from patients with metastatic colorectal cancer 16

Table S3 Characteristics of the dataset collected form patients with non-small cell lung cancer (NSCLC) 17

Table S4 The results of each evaluated schedule in patients who were identified to be initially *KRAS* wild-type 18

Table S5 Predicted progression-free-survival and time until detectable mutation in the sensitivity analysis. 20

Table S6 Parameters values of the developed model characterizing the dynamics of tumor size and mutation concentrations in NSCLC patients 21

Table S7 Parameter estimates of the tumor dynamics model based on the dataset collected form patients with NSCLC 23

Supplementary methods

1. Parameter estimate

To assist the setting of parameter values, the values of parameter describing tumor dynamics under anti-EGFR inhibitor (D_1) therapy were estimated by fitting the collected tumor sizes data[1] using the first order conditional estimation method with interaction (FOCEI) implemented in NONMEM software, version 7.4.1 (ICON Development Solutions).

A non-linear mixed-effect model was developed. Parameters were assumed to be log-normally distributed and were expressed using equation (S1). P_i represents the parameter of ith individual, P_{pop} represents typical value of the parameter, and η_i represents the random inter-individual variability (IIV) which was normally distributed with mean of 0 and variance of ω^2. The residual error was characterized with a proportional error model as is shown in equation (S2), where Obs represents observations, $IPRED$ represents individual predictions, and ε_1 represents the proportional residual error which was assumed to be normally distributed with mean of 0 and variance of ${\sigma_1}^2$.

$$P_i = P_{pop} \cdot e^{\eta_i} \qquad \text{(S1)}$$

$$Obs = IPRED \cdot (1 + \varepsilon_1) \qquad \text{(S2)}$$

Assuming the tumor growth follows an exponential growth pattern, k_{g1} was fixed as 0.03/week (=ln2/(6.8 months · 4 weeks/month)) according to a previously reported median placebo tumor doubling time colorectal carcinomas, i.e. 6.8 months (range: 3 - 24 months) [2]. k_{g2}, as was assumed, was fixed as 0.021 /week (0.03·70%). The baseline levels of TS and mutant *KRAS* (M_{ctDNA1}) were fixed according to real observations of each patient. For WT-*KRAS* patients, the baseline of T_{R1} were set to 0. For M-*KRAS* patients, the baseline of T_{R1} was estimated and the baseline of T_s equals the difference between the observed baseline and estimated baseline T_{R1}.

2. Model in a evaluation cohort

The model used in the evaluation cohort was adjusted according to the findings of the study:
1) The detectable *EGFR* L858R mutation or exon 19 deletion in ctDNA at the start of treatment indicates the tumor is sensitive to anti-EGFR inhibitor. Therefore, the sensitive clonal population (T_s) was assumed to carry one of these two mutations (M_{ctDNA1});

2) L858R mutation or exon 19 deletion became undetectable when EGFR inhibitor (D_1) started and raised back again together with the newly developed *EGFR* T790M mutation (M_{ctDNA2}) during treatment[3], which indicates the emergence of treatment resistance. Therefore the acquired resistant clonal population under D_1 (T_{R1}) was assumed to carry both M_{ctDNA1} and M_{ctDNA2};

3) A hypothetical treatment next to anti-EGFR inhibitor (D_2) was incorporated and assumed to target T790M positive NSCLC cancer (T_{R1}). In the meantime, a third mutation (M_{ctDNA3})

was able to be acquired which resulted in a third clonal population (T_{R2}) that were resistant to D_2.

More details of the model and the parameters are shown in **Supplementary Fig. S1** and **Supplementary Table S6**.

The values of parameters regarding tumor dynamics were estimated using the collected time curves of tumor sizes as described above. The residual error was characterized with an additive error model as is shown in equation S3, where Obs represents observations, $IPRED$ represents individual predictions, and ε_2 represents the additive residual error which was assumed to be normally distributed with mean of 0 and variance of ${\sigma_2}^2$. The parameter estimate results can be found in **Supplementary Table S7**.

$$Obs = IPRED + \varepsilon_2 \quad \text{(S3)}$$

References

1 Diaz, L. A., Jr. *et al.* The molecular evolution of acquired resistance to targeted EGFR blockade in colorectal cancers. *Nature* **486**, 537-540, doi:10.1038/nature11219 (2012).

2 Blagoev, K. B. *et al.* Neutral evolution of drug resistant colorectal cancer cell populations is independent of their KRAS status. *PLoS One* **12**, e0175484, doi:10.1371/journal.pone.0175484 (2017).

3 Xiong, L. *et al.* Dynamics of EGFR mutations in plasma recapitulates the clinical response to EGFR-TKIs in NSCLC patients. *Oncotarget* **8**, 63846-63856, doi:10.18632/oncotarget.19139 (2017).

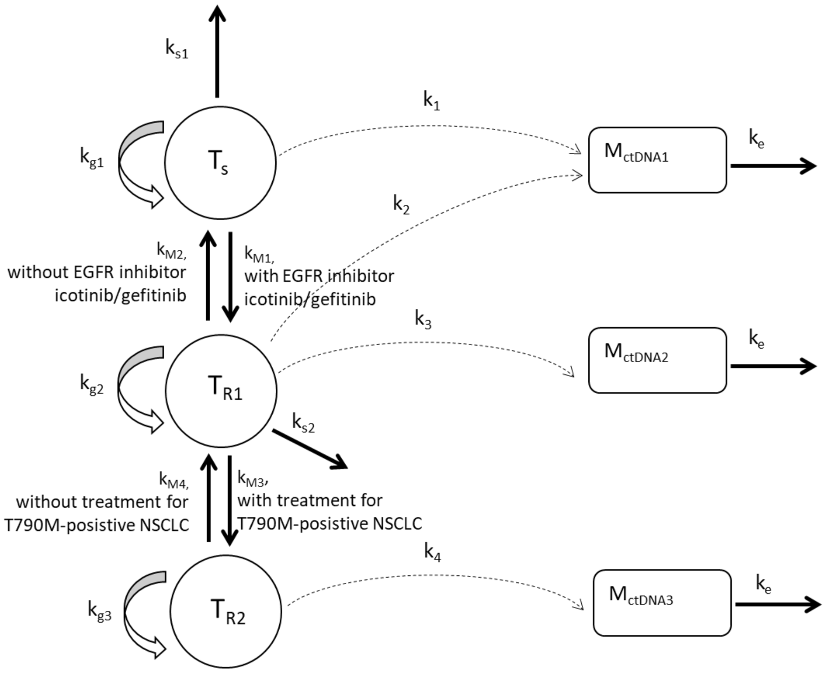

$$\frac{dT_s}{dt} = k_{g1} \cdot T_s - k_{s1} \cdot D_1 \cdot T_s - k_{M1} \cdot D_1 \cdot T_s + k_{M2} \cdot (1 - D_1) \cdot T_{R1} \quad \text{Eq. S4}$$

$$\frac{dT_{R1}}{dt} = k_{M1} \cdot D_1 \cdot T_s + k_{g2} \cdot T_{R1} - k_{s2} \cdot D_2 \cdot T_{R1} - k_{M2} \cdot (1 - D_1) \cdot T_{R1} - k_{M3} \cdot D_2 \cdot T_{R1} + k_{M4} \cdot (1 - D_2) \cdot T_{R2} \quad \text{Eq. S5}$$

$$\frac{dT_{R2}}{dt} = k_{M3} \cdot D_2 \cdot T_{R1} + k_{g3} \cdot T_{R2} - k_{M4} \cdot (1 - D_2) \cdot T_{R2} \quad \text{Eq. S6}$$

$$k_1 = k_{\max\_1} \cdot {T_s}^H / ({T_s}^H + {KT_{50}}^H) \quad \text{Eq. S7}$$

$$k_2 = k_{\max\_1} \cdot {T_{R1}}^H / ({T_{R1}}^H + {KT_{50}}^H) \quad \text{Eq. S8}$$

$$k_3 = k_{\max\_2} \cdot {T_{R1}}^H / ({T_{R1}}^H + {KT_{50}}^H) \quad \text{Eq. S9}$$

$$k_4 = k_{\max\_3} \cdot {T_{R2}}^H / ({T_{R2}}^H + {KT_{50}}^H) \quad \text{Eq. S10}$$

$$\frac{dM_{ctDNA1}}{dt} = k_1 \cdot T_S + k_2 \cdot T_{R1} - k_e \cdot M_{ctDNA1} \quad \text{Eq. S11}$$

$$\frac{dM_{ctDNA2}}{dt} = k_3 \cdot T_{R1} - k_e \cdot M_{ctDNA2} \quad \text{Eq. S12}$$

$$\frac{dM_{ctDNA3}}{dt} = k_4 \cdot T_{R2} - k_e \cdot M_{ctDNA3} \quad \text{Eq. S13}$$

$$TS = T_s + T_{R1} + T_{R2} \quad \text{Eq. S14}$$

Figure S1 The model structure that characterize the dynamics of tumor size and mutation concentrations in ctDNA from NSCLC patients.

T_s, T_{R1}, and T_{R2} represent the sizes of the three tumor clonal populations, respectively. k_{g1}, k_{g2}, k_{g3} represent the net growth rates of three clonal populations. k_{s1} and k_{s2} represent the tumor decay rate due to treatments. k_{M1} and k_{M3} represent the mutation rate constant from drug susceptible clonal population to drug resistant clonal population during the course of anti-EGFR inhibitor (D_1) and the hypothetical treatment (D_2), respectively. k_{M2} and k_{M4} represent the transition rate constant from drug resistant clonal population to drug susceptible clonal population upon the withdrawal of treatments. k_1, k_2, k_3, and k_4 represent the shedding rate constant of ctDNA which carries mutations.

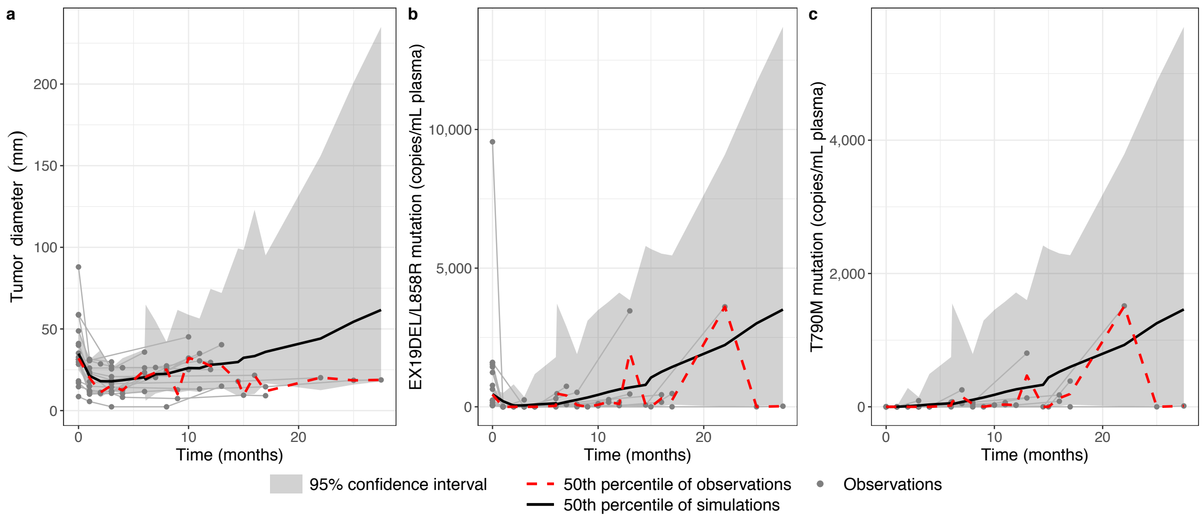

Figure S2. Model evaluation results on the time-courses of tumor diameters (a) and *EGFR* mutation concentrations including L858R mutation/ exon 19 deletion (b) and T790M mutation (c) collected from a previous clinical study where patients with non-small cell lung cancer were treated with anti-EGFR inhibitor icotinib/gefitinib.

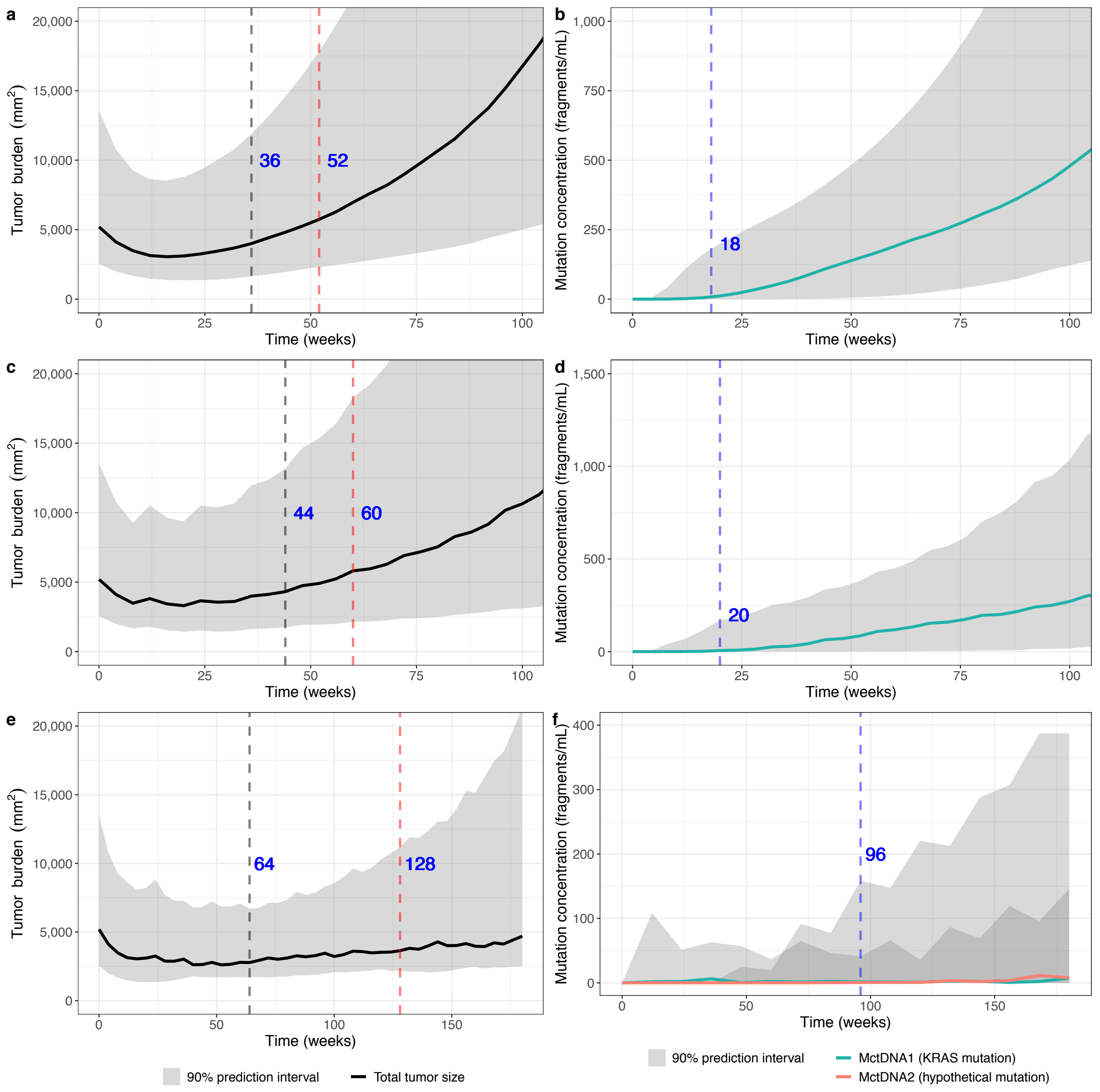

Figure S3. The simulated total tumor burden (a,c,e) and mutation concentrations (b,d,f) under continuous treatment (a,b), intermittent treatment (8-week treatment and 4-week suspension) (c,d), and adaptive treatment with the second hypothetical drug (ctDNA limits for drug adjustment: 5 and 10 fragments/ml, monitor frequency: 12 weeks) (e.f) for 100 colorectal cancer patients. Median total tumor sizes (black lines), M_{ctDNA1} (light sea green lines), and M_{ctDNA2} (salmon lines) were plotted together with corresponding 90% prediction intervals. Median PFS (black dashed vertical line), $T_{TS<TS0}$ (red dashed vertical line), and T_{mutant_test} (blue dash vertical line) were also shown in the figure.

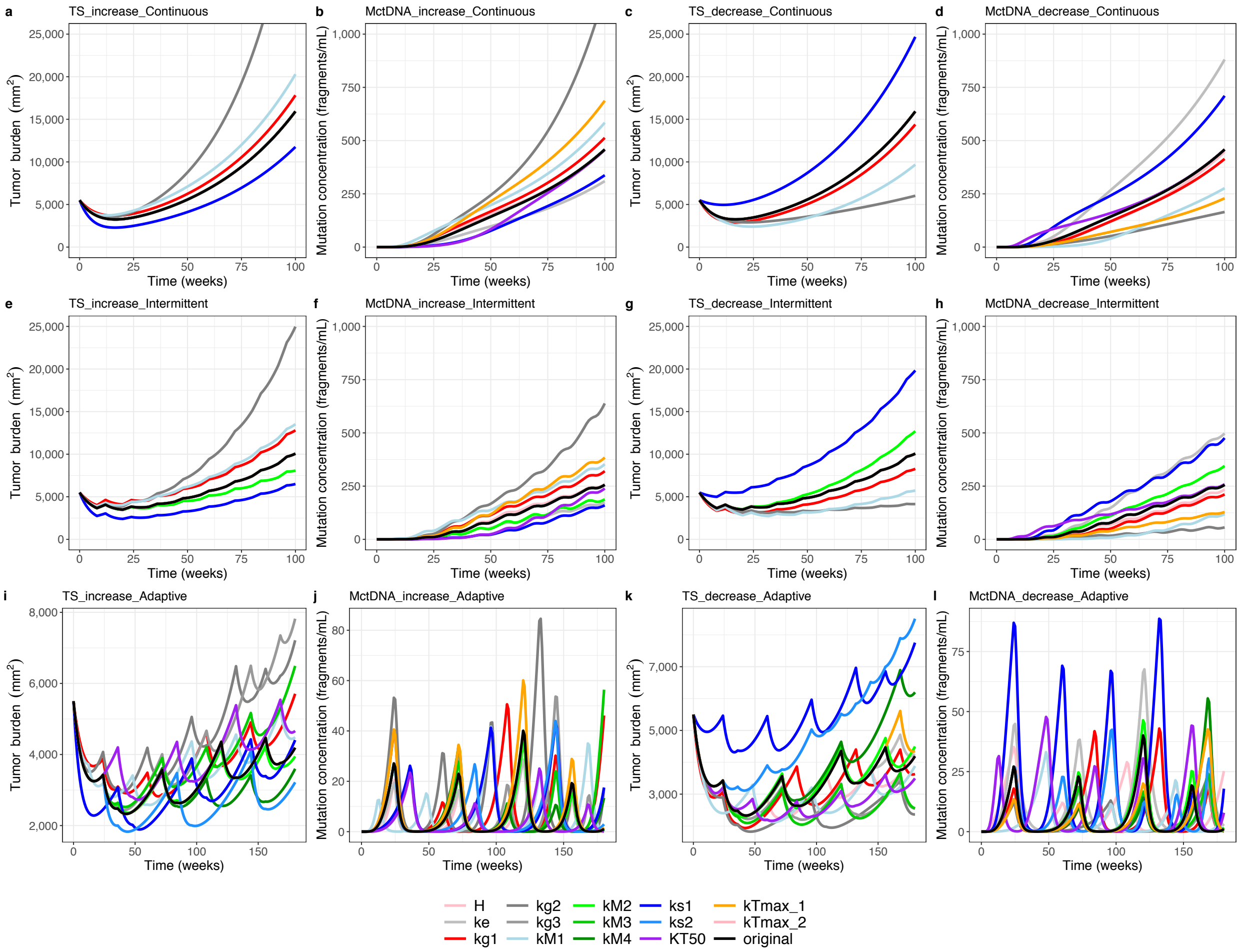

Figure S4. Simulated time curves from sensitivity analysis.

Simulated time-curves of tumor burden (a,c,e,g,i,k) and M_{ctDNA1} concentrations (b,d,f,h,j,l) based on each parameter setting under continuous treatment (a-d), intermittent treatment (8-week treatment and 4-week suspension) (e-h), and adaptive treatment (ctDNA limits for drug adjustment: 5 and 10 fragments/ml, monitor frequency: 12 weeks) (i-k). Every parameter increased by 50% (a,b,e,f,i,j) or decreased by 50% one at a time (c,d,g,h,k,l).

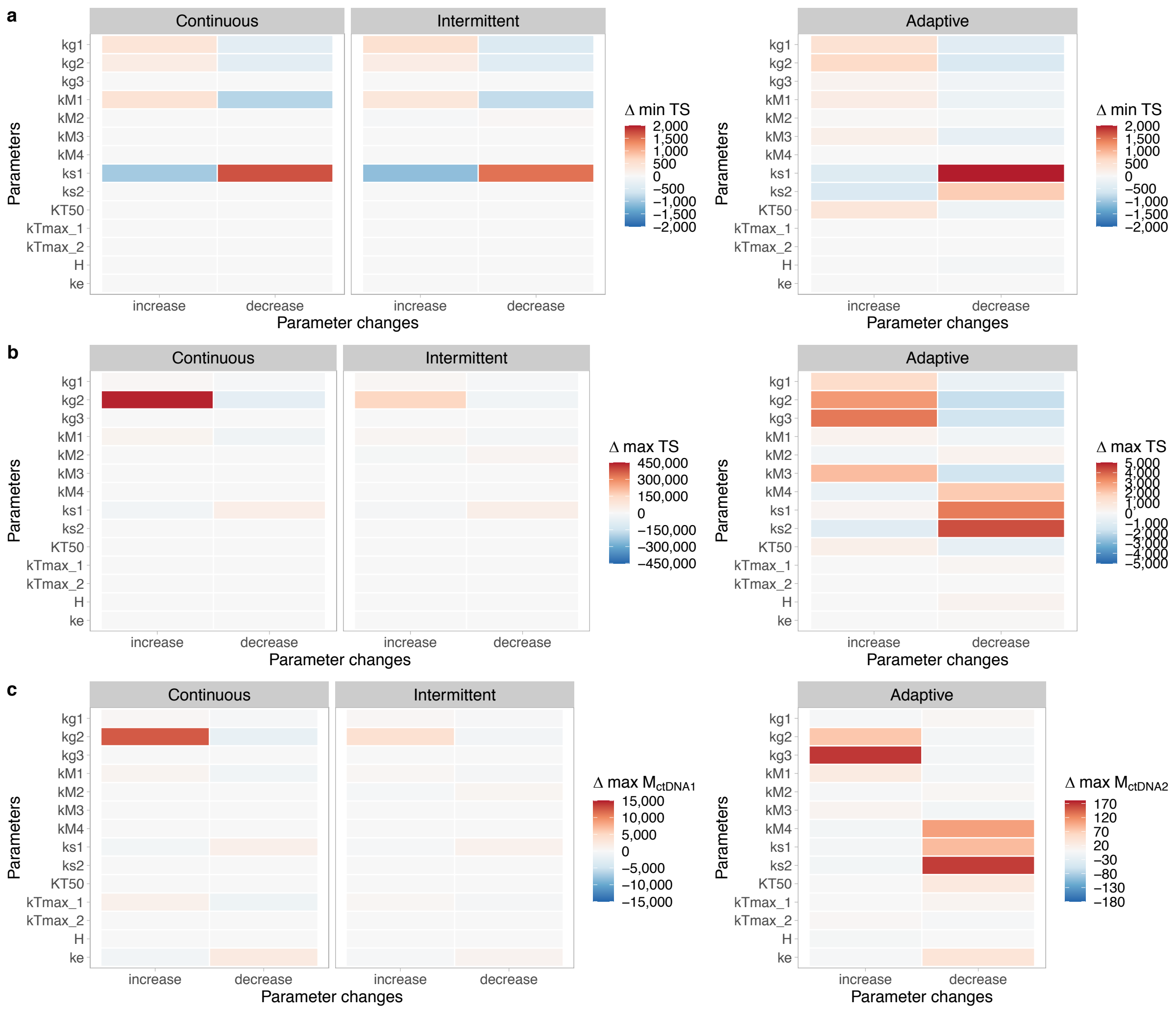

Figure S5. Relative change (Δ) of predicted minimum total tumor size (a), of total tumor size at the last simulated time point (180 weeks) (b), and of M_{ctDNA1} or M_{ctDNA2} concentrations at the last simulated time point (180 weeks) (c) compared with using original parameters in the sensitivity analysis.

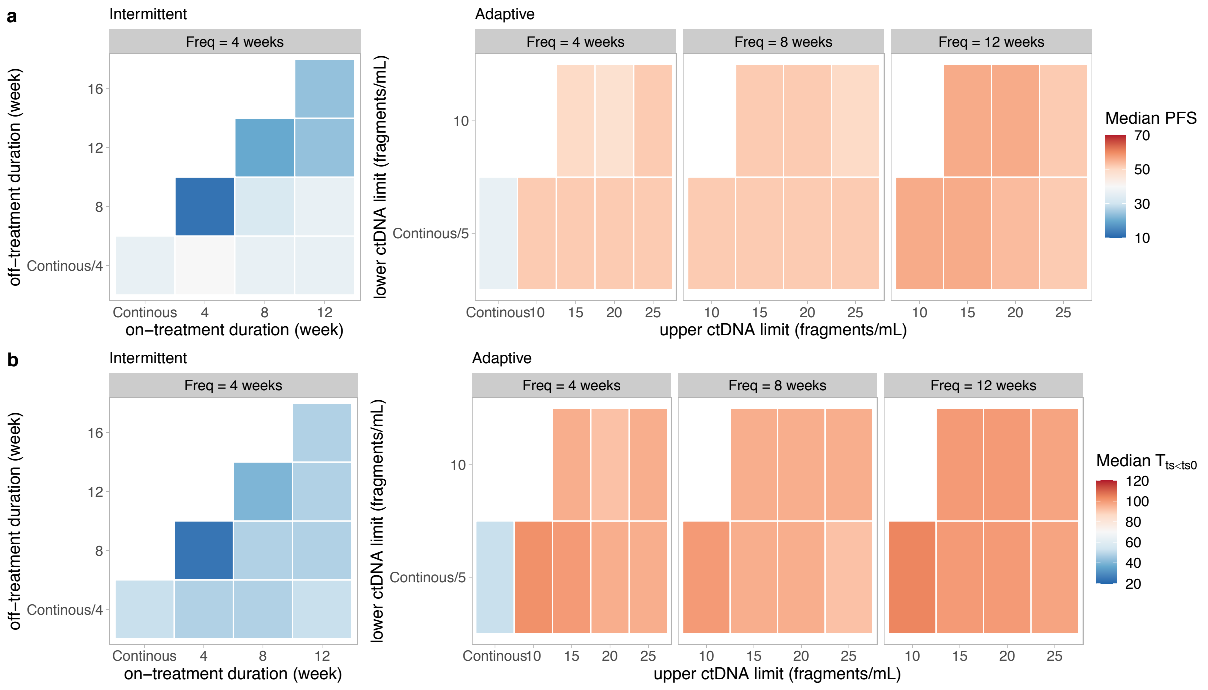

Figure S6. When fixing k_{M2} and k_{M4} to zero, the predicted median progression-free survival (PFS) (a) and the time until the tumor size had grown back to the baseline level ($T_{TS<TS0}$) (b) of evaluated regimens

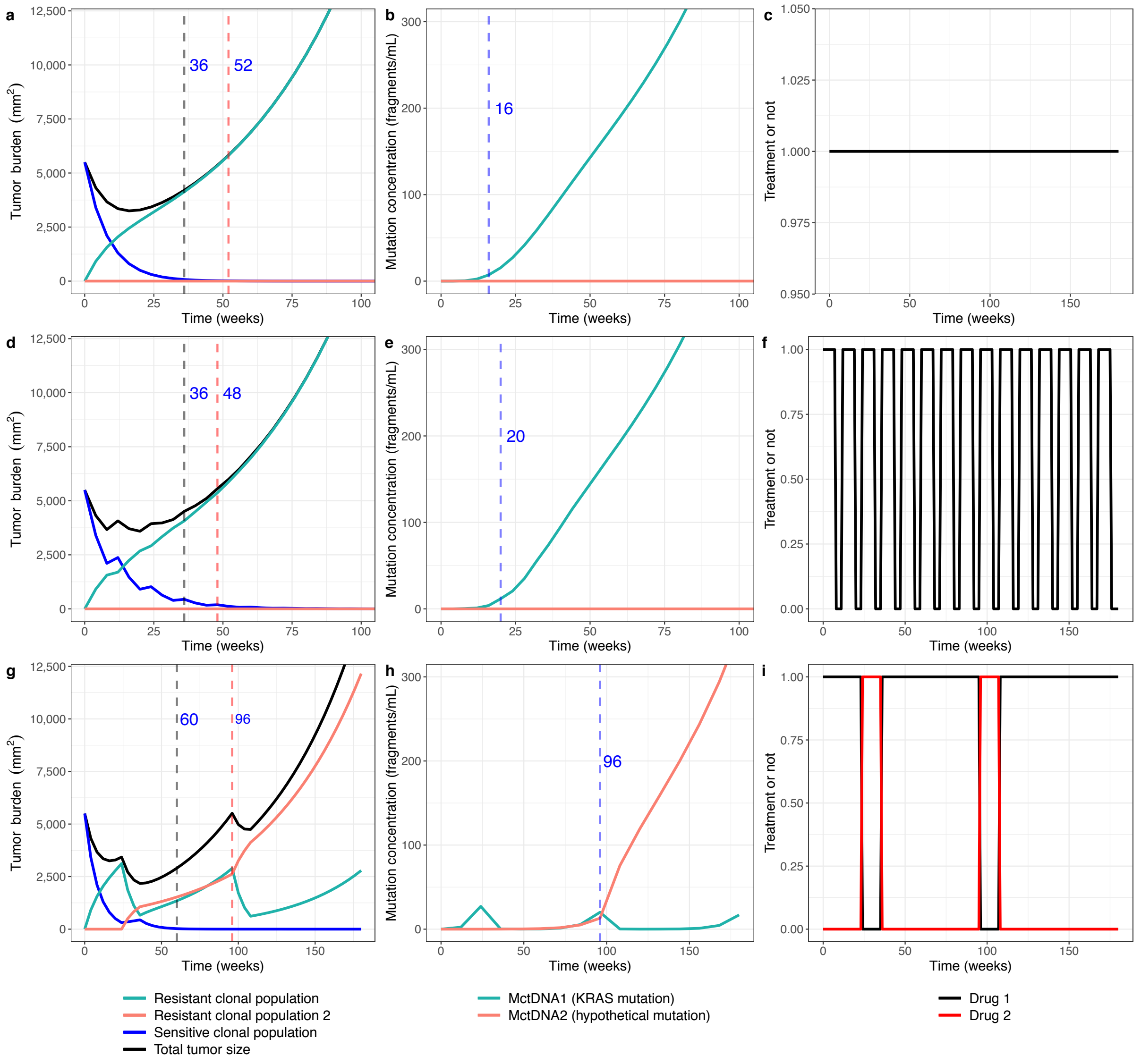

Figure S7. When fixing k_{M2} and k_{M4} to zero, the simulated time-curves of total tumor burden and each clonal population (a,d,g), mutation concentrations (b,e,h), and dosing strategies (c,f,i) of a typical subject with metastatic colorectal cancer undergoing continuous treatment (a,b,c), intermittent treatment (8-week treatment and 4-week suspension) (d,e,f), and adaptive treatment with the second hypothetical drug (ctDNA limits for drug adjustment: 5 and 10 fragments/ml, monitor frequency: 12 weeks) (g,h,i).

Estimated PFS (black dashed vertical line), $T_{TS<TS0}$ (red dashed vertical line), and T_{mutant_test} (blue dash vertical line) are also shown in the figure.

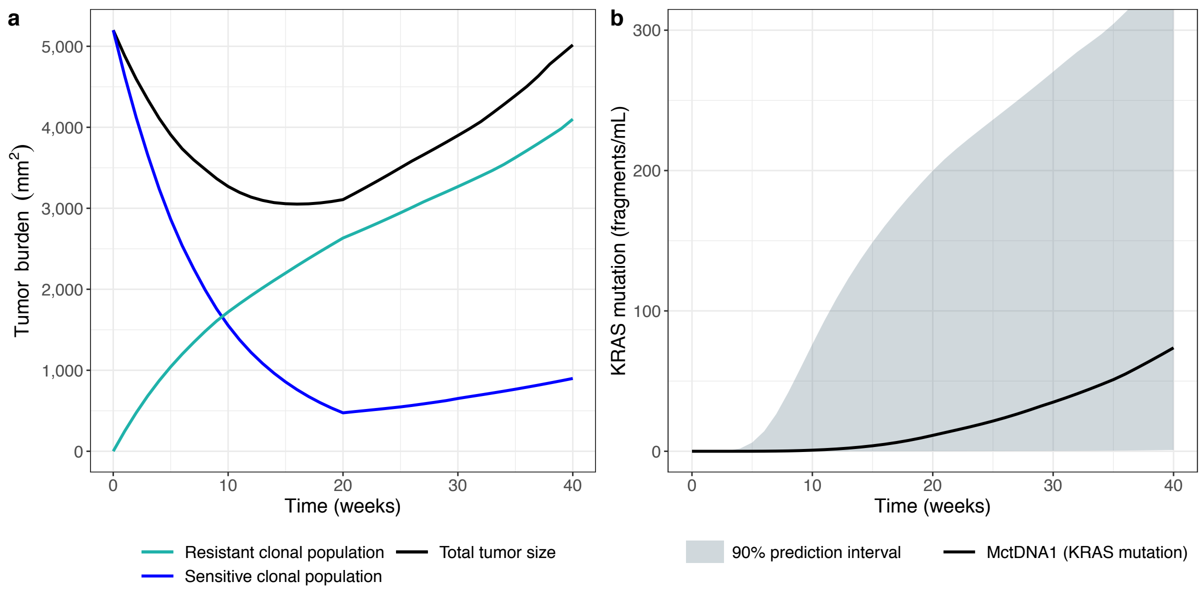

Figure S8. When fixing k_{M2} and k_{M4} to zero, model predicted total tumor burden and each clonal population (a) and mutant *KRAS* concentrations (b) under a regimen of 20-week treatment and 20-week suspension.

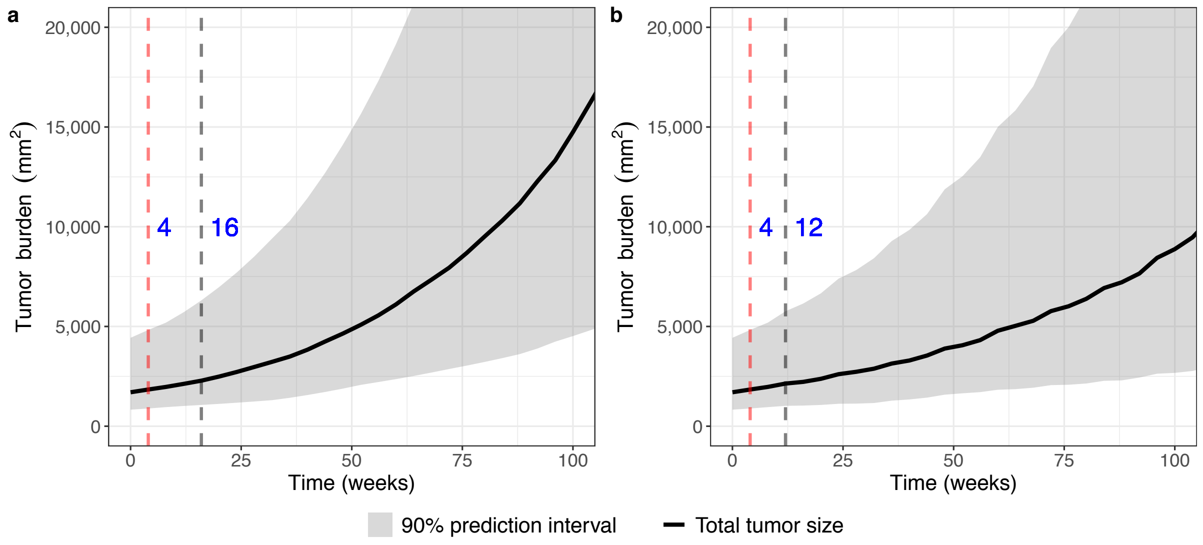

Figure S9 The simulated total tumor burden under continuous treatment (a) and intermittent treatment (8-week treatment and 4-week suspension) (b) for 100 colorectal cancer patients with detectable *KRAS* mutation pre-treatment.

Median total tumor sizes (black lines) were plotted along with 90% prediction intervals. Median PFS (black dashed vertical line) and $T_{TS<TS0}$ (red dashed vertical line) were also shown in the figure.

Table S1 Characteristics of the dataset collected from patients with metastatic colorectal cancer

	WT-*KRAS* patients	M-*KRAS* patients
Number of patients	25	3
Gender (Male (%))	15 (60%)	0 (0%)
Age (years) (median (range))	59 (42-78)	56 (48-78)
TS_0 (mm^2) (median (range))	5649 (396-38006)	1714 (1312-1849)
Baseline mutant *KRAS* (fragments/ml) (median (range))	0	411 (23-810)
PFS (week) (median (range))	23 (7-52)	7 (7-11)
Mutant *KRAS* detectable time (week) (median (range))	22 (5-34) (N=9)	0

TS_0 , baseline tumor size; PFS, progression-free survival; WT-*KRAS* patients, patients who were identified to be initially *KRAS* wild-type; M-*KRAS* patients, patients who had detectable mutant *KRAS* pre-treatment.

Table S2 Parameter estimates of the tumor dynamics model based on the dataset collected from patients with metastatic colorectal cancer

**Parameters**	**Estimate (RSE)**	**IIV (CV%) [shrinkage]**
WT-*KRAS* patients		-
T_{S_0} (mm^2)	TS_{Obs} (fixed)	-
T_{R1_0} (mm^2)	0 (fixed)	-
M-*KRAS* patients		
T_{S_0} (mm^2)	$TS_{Obs} - T_{R1\_0\_Est}$	-
T_{R1_0} (mm^2)	1830 (17%)	0 (fixed)
k_{g1} (/week)	0.03 (fixed)	68.6% [14%]
k_{g2} (/week)	$0.7 \cdot k_{g1}$ (fixed)	-
k_{s1} (/week)	0.127 (5%)	-
k_{M1} (/week)	0.0459 (18%)	-
Residual error		-
Prop (CV%)	21.7% (11%)	

TS_{Obs}, observed total tumor size, $T_{R1\_0\_Est}$, estimated baseline of T_{R1}, WT-*KRAS* patients, patients who were identified to be initially *KRAS* wild-type; M-*KRAS* patients, patients who had detectable mutant *KRAS* pre-treatment. RSE, relative standard error, CV, coefficient of variation, IIV, inter-individual variability, Prop, proportional residual error.

Relative standard errors (RSEs) of parameter estimates were all within an acceptable range (<30%).

Table S3 Characteristics of the dataset collected form patients with non-small cell lung cancer (NSCLC)

	**Values**
Number of patients	16
TS_0 (mm) (median (range))	33.92 (16.97-87.96)
Baseline EGFR L858R mutation /exosome 19 deletion concentration (copies/ml) (median (range))	438.75 (42-9555.56)
PFS (months) (median (range))	12 (4-25)
EGFR T790M mutation detectable time (months) (median (range))	10.5 (3-27.5)

TS_0, baseline tumor size; PFS, progression-free survival

Table S4 The results of each evaluated schedule in patients who were identified to be initially *KRAS* wild-type

**Schedules**			**Median PFS* (90% interval) (weeks)**	**Median** $T_{TS<TS0}$ **(90% interval) (weeks)**	**Median** T_{mutant_test} **(90% interval) (weeks)**
Continuous schedule (standard of care)			36 (32-44)	52 (36-72)	18 (8-52.6)
Intermittent schedules<br>D_1 was administered for N weeks and suspended for M weeks. Total treatment time was 180 weeks.					
N (weeks)	M (weeks)				
4	4		48 (24-80)	56 (24-112.8)	28 (8-92.8)
4	8		12 (12-72.6)	24 (12-96)	32 (8-112.4)
8	4		44 (32-60)	60 (36-104.2)	20 (8-80)
8	8		38 (16-64)	60 (32-113.2)	24 (8-93.2)
8	12		20 (16-20)	40 (20-116.4)	28 (8-108)
12	4		40 (32-56)	60 (36-92.2)	24 (8-68.4)
12	8		40 (20-60)	60 (36-112.2)	28 (8-88)
12	12		24 (20 -49.0)	64 (24-116.2)	32 (8-100.4)
12	16		24 (20-28)	52 (24-108.4)	32 (8-96)
Adaptive schedules with a hypothetical second treatment<br>D_1 was continuously given, and suspended and switched to D_2 when the ctDNA measurement increased to higher than UP fragment/ml. Treatment switched back to D_1 when ctDNA measurement decreased back to lower than LOW fragment/ml. Total treatment time was 180 weeks.					
LOW (fragment/ml)	UP (fragment/ml)	Monitoring frequency of ctDNA (weeks)			
5	10	4	62 (36-118.4)	124 (45.2-170.8)	100 (36-169.2)
5	15	4	60 (32-116.4)	132 (45.8-176)	108 (36-172)
5	20	4	60 (32-112.4)	124 (46.8-180)	102 (36-168)
5	25	4	60 (32-112.4)	120 (44-172.4)	102 (36-168.2)
10	15	4	56 (32-124)	124 (47.2-172.8)	108 (42.4-172.8)
10	20	4	56 (32-108.4)	120 (47.8-172.2)	108 (44-176)
10	25	4	56 (32-108.8)	114 (44-172.2)	110 (44-175.8)
5	10	8	60 (32-112.4)	120 (44.8-	96 (40-163.2)

				163.2)	
5	15	8	60 (32-104.8)	120 (44-169)	96 (40-170.4)
5	20	8	56 (32-100.4)	120 (44-168)	96 (40-168)
5	25	8	56 (32-104.4)	116 (44.8-168)	96 (40-168)
10	15	8	60 (32-104.4)	120 (44-168)	96 (45.6170.4)
10	20	8	56 (32-108.4)	120 (44-164.4)	96 (45.6-168)
10	25	8	56 (32-104.8)	118 (44.2-160)	104 (47.2-168)
5	10	12	64 (32-108)	128 (44-164)	96 (48-180)
5	15	12	60 (36-120)	124 (44-176)	102 (48-168)
5	20	12	60 (32-104.2)	120 (44-157.6)	108 (48-180)
5	25	12	60 (32-104)	120 (44-156)	108 (48-168)
10	15	12	62 (32-108.2)	124 (44-176)	102 (48-168)
10	20	12	60 (32-108)	120 (44-157.6)	108 (48-168)
10	25	12	60 (36-104.2)	120 (44-156)	108 (48-168)

*Disease progression was defined by WHO criteria

D_1, anti-EGFR inhibitor; D_2, a hypothetical second treatment to which the newly acquired clone is susceptible; PFS, Progression-free survival; T_{mutant_test} , time until detectable mutation; $T_{TS<TS0}$, the time until the tumor size had grown back to the baseline level; ctDNA, circulating tumor DNA

Table S5 Predicted progression-free-survival and time until detectable mutation in the sensitivity analysis.

	Continuous schedule		Intermittent schedule (8-week treatment and 4-week suspension)		Adaptive schedule (ctDNA limits for drug adjustment: 5 and 10 fragments/ml, monitor frequency 12 weeks)	
	**PFS (weeks) (Relative change*)**					
**Parameters**	**Increase 50%**	**Decrease 50%**	**Increase 50%**	**Decrease 50%**	**Increase 50%**	**Decrease 50%**
k_{g1}	-	-	36 (-8)	48 (+4)	96 (+32)	-
k_{g2}	28 (-8)	52 (+16)	32 (-12)	84 (+40)	56 (-8)	76 (+12)
k_{g3}	-	-	-	-	60 (-4)	-
k_{s1}	-	-	-	32 (-12)	36 (-28)	60 (-4)
k_{s2}	-	-	-	-	-	60 (-4)
k_{M1}	32 (-4)	44 (+8)	36 (-8)	60 (+16)	84 (+20)	44 (-20)
k_{M2}	-	-	48 (+4)	40 (-4)	-	60 (-4)
k_{M3}, k_{M4}	-	-	-	-	-	-
k_e	-	-	-	-	-	-
H	-	-	-	-	-	96 (+32)
KT_{50}	-	-	-	-	36 (-28)	84 (+20)
$k_{\max\_1}$	-	-	-	-	-	-
$k_{\max\_2}$	-	-	-	-	-	-
	**T_{mutant_test} of M_{ctDNA1} (weeks) (Relative change*)**				**T_{mutant_test} of M_{ctDNA2} (weeks) (Relative change*)**	
**Parameters**	**Increase 50%**	**Decrease 50%**	**Increase 50%**	**Decrease 50%**	**Increase 50%**	**Decrease 50%**
k_{g1}	-	20 (+4)	-	24 (+4)	120 (-48)	180 (+12)
k_{g2}	-	20 (+4)	-	28 (+8)	108 (-60)	No result
k_{g3}	-	-	-	-	120 (-48)	No result
k_{s1}	24 (+8)	12 (-4)	32 (+12)	16 (-4)	No result	72 (-96)
k_{s2}	-	-	-	-	No result	108 (-60)
k_{M1}	12 (-4)	32 (+16)	16 (-4)	44 (+24)	144 (-24)	No result
k_{M2}	-	-	-	-	-	132 (-36)
k_{M3}	-	-	-	-	120 (-48)	No result
k_{M4}	-	-	-	-	No result	132 (-36)
k_e	-	-	-	-	-	132 (-36)
H	20 (+4)	12 (-4)	28 (+8)	16 (-4)	-	120 (-48)
KT_{50}	28 (+12)	8 (-8)	32 (+12)	8 (-12)	180 (+12)	96 (-72)
$k_{\max\_1}$	-	20 (+4)	-	24 (+4)	-	180 (+12)
$k_{\max\_2}$	-	-	-	-	132 (-36)	-

PFS, Progression-free survival; T_{mutant_test} , time when mutation concentration became detectable; M_{ctDNA1}, *KRAS* mutation; M_{ctDNA2}, the second hypothesis mutation; -, result same as that under the original parameter setting; No result, the mutant gene concentrations did not reach the detectable limit (5 fragments/ml) by the end of simulation time (180 week).

*With original parameters, the predicted PFS were 36, 44, and 64 weeks under continuous, intermittent, and adaptive schedule, respectively, and the predicted T_{mutant_test} were 16, 20, and 168 weeks, respectively

Table S6 Parameters values of the developed model characterizing the dynamics of tumor size and mutation concentrations in NSCLC patients

Parameters	Description	Typical Values	Ref.
T_{s_0} (mm)	Baseline of T_s	35	Data
T_{R1_0} (mm)	Baseline of T_{R1}	0	Mutation was assumed to be acquired during treatment
T_{R2_0} (mm)	Baseline of T_{R2}	0	
M_{ctDNA1_0} (copies/ml)	Baseline of *EGFR* L858R mutation or exon 19 deletion (M_{ctDNA1})	450	
M_{ctDNA2_0} (copies/ml)	Baseline of *EGFR* T790M mutation (M_{ctDNA2})	0	
M_{ctDNA3_0} (copies/ml)	Baseline of a third hypothetical mutation (M_{ctDNA3})	0	
k_{g1} (/month)	Growth rate constant of T_s	0.07	Estimated
k_{g2} (/month)	Growth rate constant of T_{R1}	0.049	
k_{g3} (/month)	Growth rate constant of T_{R2}	0.035	
k_{s1} (/month)	Tumor shrinkage rate constant due to D_1 (anti-EGFR inhibitor)	0.8	Estimated
k_{s2} (/month)	Tumor shrinkage rate constant due to D_2 (the second hypothetical treatment)	0.8	k_{s1}
k_{M1} (/month)	Mutation rate from T_s to T_{R1} when D_1=1	0.6	Estimated
k_{M2} (/month)	Mutation rate from T_{R1} to T_s when D_1=0	0.4	Lower than k_{M1}
k_{M3} (/month)	Mutation rate from T_{R1} to T_{R2} when D_2=1	0.6	k_{M1}
k_{M4} (/month)	Mutation rate from T_{R2} to T_{R1} when D_2=0	0.4	k_{M2}
H	Hills coefficient	5	Visually matching the slope of T790M mutation time-courses
KT_{50} (mm)	The size of tumor that provide half-maximal shedding rate of ctDNA	30	
$k_{\max\_1}$ (copies /ml/(month*mm))	Maximum shedding rate of M_{ctDNA1}	120	
$k_{\max\_2}$ (copies /ml/(month*mm))	Maximum shedding rate of M_{ctDNA2}	50	
$k_{\max\_3}$ (copies /ml/(month*mm))	Maximum shedding rate of M_{ctDNA3}	50	

k_e (/month)	ctDNA eliminate rate constant	2
IIV_B (ω_1)	Standard deviation of IIV of baselines	0.6
IIV_k_g (ω_2)	Standard deviation of IIV of k_g	0.2

ctDNA, circulating tumor DNA; IIV, inter-individual variability

Table S7 Parameter estimates of the tumor dynamics model based on the dataset collected form patients with NSCLC

**Parameters**	**Estimate (RSE%)**	**IIV (CV%) [shrinkage]**
T_{s_0} (mm)	TS_{0_Obs} (fixed)	
T_{R1_0} (mm)	0 (fixed)	
k_{g1} (/month)	0.0675 (45%)	105.4% [6%]
k_{g2} (/month)	$0.7 \cdot k_{g1}$ (fixed)	-
k_{s1} (/month)	0.835 (23%)	74% [3%]
k_{M1} (/month)	0.553 (28%)	-
Residual error		-
Add (mm)	2.67 (34%)	

RSE, relative standard error; CV, coefficient of variation; IIV, inter-individual variability; Add, additive residual error
